# An Up-to-Date Review on the Remediation of Dyes and Phenolic Compounds from Wastewaters Using Enzymes Immobilized on Emerging and Nanostructured Materials: Promises and Challenges

**DOI:** 10.3390/nano13152152

**Published:** 2023-07-25

**Authors:** Mohammed K. Al-Sakkaf, Ibrahim Basfer, Mustapha Iddrisu, Salem A. Bahadi, Mustafa S. Nasser, Basim Abussaud, Qasem A. Drmosh, Sagheer A. Onaizi

**Affiliations:** 1 Department of Chemical Engineering, King Fahd University of Petroleum and Minerals, Dhahran 31261, Saudi Arabia; 2 Gas Processing Center, College of Engineering, Qatar University, Doha 2713, Qatar; 3 Department of Materials Science and Engineering, King Fahd University of Petroleum and Minerals, Dhahran 31261, Saudi Arabia; 4 Interdisciplinary Research Center for Hydrogen and Energy Storage, King Fahd University of Petroleum and Minerals, Dhahran 31261, Saudi Arabia

**Keywords:** enzyme immobilization, graphene, carbon nanotubes (CNTs), metal–organic frameworks (MOFs), wastewater treatment, dye and phenolic compound remediation

## Abstract

Addressing the critical issue of water pollution, this review article emphasizes the need to remove hazardous dyes and phenolic compounds from wastewater. These pollutants pose severe risks due to their toxic, mutagenic, and carcinogenic properties. The study explores various techniques for the remediation of organic contaminants from wastewater, including an enzymatic approach. A significant challenge in enzymatic wastewater treatment is the loss of enzyme activity and difficulty in recovery post-treatment. To mitigate these issues, this review examines the strategy of immobilizing enzymes on newly developed nanostructured materials like graphene, carbon nanotubes (CNTs), and metal–organic frameworks (MOFs). These materials offer high surface areas, excellent porosity, and ample anchoring sites for effective enzyme immobilization. The review evaluates recent research on enzyme immobilization on these supports and their applications in biocatalytic nanoparticles. It also analyzes the impact of operational factors (e.g., time, pH, and temperature) on dye and phenolic compound removal from wastewater using these enzymes. Despite promising outcomes, this review acknowledges the challenges for large-scale implementation and offers recommendations for future research to tackle these obstacles. This review concludes by suggesting that enzyme immobilization on these emerging materials could present a sustainable, environmentally friendly solution to the escalating water pollution crisis.

## 1. Introduction

Environmental pollution has become a major global concern, with a wide range of contaminants, such as phenolic compounds and organic dyes, posing significant threats to water quality and ecosystem health [1,2,3,4]. The increasing release of these toxic and recalcitrant substances into the environment, originating from various industrial wastewater streams such as textile, pharmaceutical, and petrochemical industries, has caused widespread concern [5,6]. Contaminated water sources can lead to adverse effects on aquatic life and have potentially detrimental consequences for human health, as these pollutants may exhibit carcinogenic, mutagenic, or teratogenic effects [7]. In particular, understanding the effect of organic dyes and phenolic compounds on human health is crucial for the development of effective policies and remediation strategies to minimize the risks associated with these contaminants [8]. As the demand for clean water continues to increase due to population growth and industrialization, there is an urgent need to develop effective, efficient, and sustainable strategies for environmental remediation, focusing on the removal of these pollutants from wastewater [8,9,10].

Numerous conventional techniques have been employed to treat phenolic wastewater and organic dyes, each with its own advantages and disadvantages. For instance, adsorption is simple, flexible, and can be highly efficient with low capital cost, but the performance is adsorbent-dependent, and some adsorbents can be expensive [11,12,13,14,15,16,17]. Distillation, while effective, can be energy-intensive and may not be suitable for all types of contaminants [18,19,20,21]. Chemical oxidation can be highly effective, but it often involves the use of harsh chemicals that can be harmful to the environment [22,23,24]. Extraction is a fast and simple process, but it requires highly selective solvents, which can be expensive and harmful to the environment [25,26,27,28]. Membrane separation is easy to operate with high selectivity, but it can be expensive and suffers from issues such as membrane fouling [29,30,31,32]. Photocatalytic oxidation is a promising technique, but it often requires specific conditions to be effective [33,34,35,36].

Given these limitations of conventional techniques, there has been growing interest in environmental protection through the effective treatment of polluting streams [37,38,39,40], the utilization of sustainable and biodegradable materials [41,42,43,44,45,46,47,48], and the application of microorganisms for the biological treatment of wastewater contaminated with organic dyes, and phenolic compounds have also been explored [49,50]. Microorganisms utilize various enzymatic systems for the oxidative transformation of organic molecules, including laccases, ligninases, tyrosinases, monooxygenases, and dioxygenases [51]. Flavoenzymes, known as azoreductases, are present in both microorganisms and higher eukaryotes and are involved in the detoxification and biotransformation of azo dyes [52]. Furthermore, microorganisms utilize specific intracellular enzymes, namely oxidoreductases, to catalyze the metabolism of phenolic compounds [53]. These enzymatic systems offer several advantages over conventional techniques, including cost-effectiveness, sustainability, and the ability to operate under mild conditions [54,55,56]. This highlights the critical function of enzymes in the biodegradation processes of various organic pollutants, demonstrating the potential of harnessing these enzymatic systems for efficient and eco-friendly pollutant removal strategies [56]. As a result, a new approach has arisen in recent times where extracellular enzymes are utilized instead of whole microbial cells for the remediation of wastewater contaminated with organic substances.

Enzymes act as highly effective biological catalysts that enable specific reactions to occur. The lock and key model or the induced fit model can be used to explain their efficiency. By reducing the activation energy and stabilizing the transition state, enzymes enhance the reaction rate [57,58]. Enzymes possess desirable qualities such as high efficiency, high selectivity, and the ability to operate under milder conditions compared to other chemical catalysts. Enzymes offer a cost-effective advantage in that they operate under mild conditions, eliminating the need for expensive equipment that would otherwise be required for chemical catalysts to achieve extreme conditions such as high pressure or temperature. Their natural origin also makes them environmentally friendly due to their biodegradability and low environmental impact.

There has been growing interested in employing enzymes for the treatment of dye wastewater, and previous research has examined the use of various enzymes, including soybean peroxidase [59,60], horseradish peroxidase (HRP), lignin peroxidase (LiP) [61], and laccase [62,63], for their potential in treating dyes. Peroxidase, a member of the oxidoreductase enzyme family, can enable the oxidation of diverse substances in the presence of an oxidizing agent like chlorine, hydrogen peroxide, and potassium permanganate. A significant use of peroxidase is its ability to degrade aromatic compounds, especially synthetic dyes. This occurs when they are decomposed into individual components and the oxidative polymerization of phenolic compounds is triggered, leading to the creation of insoluble polymers [58,64]. Hydrogen peroxide plays a crucial role in the catalytic cycle of peroxidase enzymes. The reaction begins with the reaction between the Fe(III) state of peroxidase and hydrogen peroxide, leading to the formation of a high-oxidation-state intermediate consisting of a cation radical based on porphyrin and an Fe(IV) oxo ferryl center [65]. After the initial oxidation, the process consists of two reduction steps that bring the peroxidase back to its original state, compound II, with the production of free radicals. These free radicals then undergo polymerization. However, a high concentration of hydrogen peroxide may inhibit the process, leading to a decrease in enzymatic activity [66].

The application of enzymes for dye wastewater treatment at an industrial scale is often hindered by various limitations such as elevated production expenses, reduced long-term operational stability, restricted reusability, and limited shelf life after the initial use [59,67]. In their crude form, enzymes may exhibit limited catalytic activity because of their vulnerability to inhibition, particularly in the case of complex dye wastewater [68]. Heavy metals can have a detrimental effect on enzymatic activity, as different enzymes show varying degrees of sensitivity to these substances [58]. In certain cases, heavy metals, such as mercury, can react with the reactive groups present in enzymes and render them incapable of catalyzing further reactions [69]. As the complexity of wastewater effluent increases, it is expected that enzyme activity will decline, further underscoring the challenges associated with the use of enzymes in the treatment of wastewater [70,71].

In recent years, the use of free enzymes has gained considerable attention among the various proposed methods for removing phenolic compounds [72,73,74,75]. Enzymatic treatment offers a sustainable and eco-friendly alternative to conventional physicochemical treatment methods, such as adsorption, coagulation, and advanced oxidation processes [76,77]. Free enzymes are biodegradable, highly efficient, and selective biological catalysts that can operate under mild conditions, thus reducing energy consumption and minimizing the generation of harmful byproducts [78,79].

Researchers have explored the technique of enzyme immobilization as a means to overcome limitations that free enzymes encounter, including reduced performance, high costs, and impracticality for large-scale applications [56,80,81]. Enzymes have limited operational stability, which can negatively impact their catalytic efficiency. Factors like temperature, pH, and exposure to harsh conditions or solvents can lead to enzyme denaturation, degradation, or aggregation, thereby limiting their effectiveness [82]. Enzyme immobilization can improve the stability of enzymes as it offers a physical support system that safeguards them against destabilizing agents while maintaining their original structure [80,83]. The recovery and reuse of free enzymes after a reaction can be time-consuming and costly, but enzyme immobilization allows for easy separation from the reaction mixture and enables reuse for several reaction cycles, thereby decreasing the overall costs of enzyme usage [84,85,86]. In some reactions, free enzymes can experience reduced catalytic activity due to mass transfer limitations, substrate and product inhibition, or poor substrate solubility in water. Immobilization can overcome these challenges by creating custom biocatalytic systems with improved mass transfer properties, enhanced enzyme-substrate interactions, and optimized reaction conditions [54]. Moreover, free enzymes may exhibit low selectivity in certain reactions, especially when working with chiral compounds or complex substrate mixtures. Immobilization has the potential to enhance selectivity by enabling precise control over enzyme orientation and creating a microenvironment that promotes selective catalysis [87,88].

According to Nguyen et al. [89], immobilized enzymes are more effective in eliminating phenolic compounds than free enzymes due to the synergistic effect of enzymatic reactions and pollutant adsorption on the solid support. While not examining the competition between the adsorption of pollutants and products or the influence of product adsorption on enzyme activity, previous research has demonstrated that the adsorption capacity of the support medium deteriorates after multiple applications, even in the absence of enzymes. This suggests that irreversible adsorption of pollutants or products could be involved in the overall process, particularly when immobilized enzymes are recycled. Nguyen et al. [89] suggested that addressing the removal of irreversibly adsorbed pollutants or products could potentially enhance the effectiveness of the immobilized enzyme system.

There is a broad range of nanomaterials that are widely employed for the purpose of enzyme immobilization. This includes but is not limited to metal oxides [90], carbon dots [91], covalent organic frameworks (COFs) [92], graphene [93], CNTs [94], and MOFs [95]. Each of these nanomaterials possesses unique properties that make them suitable for enzyme immobilization. They often exhibit a high surface area to volume ratio, excellent conductivity, good chemical stability, and strong adsorption capabilities, which make them advantageous in improving the performance of immobilized enzymes [96].

Metal oxides like titanium dioxide and zinc oxide offer robustness, chemical stability, and biocompatibility, making them useful for enzyme immobilization [90,97]. Carbon dots, with their superior optical properties and biocompatibility, also have applications in this area [91]. COFs, due to their designable structures, large pore size, and high surface area, provide ideal platforms for enzyme immobilization [92]. However, this review specifically focuses on the unique advantages of graphene, CNTs, and MOFs for enzyme immobilization. Graphene and its derivatives, such as graphene oxide (GO), are widely used for enzyme immobilization due to their high surface area, excellent thermal and electrical conductivity, and strong π-π stacking interactions, which allow effective enzyme adsorption and retention of their bioactivity [83,98]. CNTs offer similar benefits, with additional advantages coming from their tubular structure, which provides a protective environment for enzymes, enhancing their stability and reusability [99,100]. MOFs, with their highly ordered structures and large surface areas, offer unique possibilities for enzyme immobilization. Their pore size, shape, and functionality can be finely tuned, allowing the accommodation of a wide range of enzymes while preserving their activity and stability [101,102]. 

In this article, we review the advancements made in developing supports for enzyme immobilization and the application of these biocatalytic materials in the removal of dyes and phenolic contaminants from polluted waters. Specifically, this review underscores the use of emerging materials, such as graphene-based materials, CNTs, and MOFs, as appealing supports for enzyme immobilization and the subsequent applications of enzymes immobilized on these nanostructured materials in treating wastewater containing dyes and phenolic pollutants. We also address the limitations and challenges associated with implementing this wastewater treatment technology on a larger scale and provide recommendations to overcome these obstacles. This review article distinguishes itself from other published works by its specific emphasis on the potential applications of effective materials for environmental remediation (dyes and phenolic compounds), in contrast to more general review articles on enzyme immobilization.

## 2. Enzyme Immobilization Techniques

Enzyme immobilization is a technique to improve enzyme stability and reusability while maintaining their activity. It refers to the physical or chemical confinement of enzymes in a distinct phase different from the substrate’s phase [103]. These techniques can be classified into two broad categories: physical and chemical methods [103,104]. In the following subsections, we delve deeper into each of these techniques, providing a detailed review of their principles, advantages, and practical applications. 

### 2.1. Physical Techniques

Physical immobilization, as the earliest form of immobilization, only involves physical interactions. In this method, neither the immobilizer nor the immobilization agent is changed, linked, or modified. This technique includes encapsulation, entrapment, and adsorption. These processes do not necessitate a covalent bond between the enzyme and the support, therefore maintaining the enzyme’s native structure [105]. Adsorption involves enzymes interacting with a support material through forces such as hydrophobic interactions or salt bridges, while entrapment is a technique where enzymes are confined within gels or fibers using covalent or non-covalent bonds. Similarly, encapsulation secures enzymes within semi-permeable capsules, allowing for the movement of small substrates or products while restricting the migration of larger enzymes [104,105,106].

#### 2.1.1. Adsorption

Enzymes can be adsorbed onto support materials through interactions such as hydrophobic forces and salt bridges. Enzyme adsorption onto the support physically can be achieved by immersing the support material in the enzyme solution or by drying enzymes onto electrode surfaces. This immobilization method protects the adsorbed enzymes from factors such as proteolysis, aggregation, and interaction with hydrophobic surfaces [107]. Scientists have utilized eco-friendly materials as enzyme supports to promote sustainable practices. For instance, coconut fibers can retain high amounts of water and have strong cation exchange properties, microcrystalline cellulose has a strong binding capacity, and kaolin offers good enzyme retention through micro/mesoporous materials and chemical acetylation with thiol functionalization and large surface areas that are suitable for redox reactions [108,109,110,111,112,113]. Silanized molecular sieves have been found to be an effective support for enzyme adsorption, owing to the presence of silanols on the surface of the pores that allow for enzyme immobilization through the process of hydrogen bonding [114]. Modifications to the current support materials could potentially enhance enzyme immobilization. Prior investigations have delineated the water activity patterns of polypropylene hydrophobic granules-bound lipase, notably Accurel EP-100 [115]. It was observed that reducing the particle size of Accurel has a positive effect on reaction rates and enantiomeric ratios during biocatalysis [116]. 

To improve both process control and the cost-effectiveness of production, the immobilization of Yarrowia lipolytica lipase on supports like octadecyl-sepabeads and octyl-agarose through physical adsorption has been explored. As a result of this process, there were significant improvements in yields and a tenfold increase in stability when compared to free lipase. Octadecyl-sepabeads, which are hydrophobic in nature, enhance the affinity between the enzyme and support, explaining this observation [117]. After being adsorbed onto biodegradable poly (3-hydroxybutyrate-co-hydroxyvalerate), Candida rugosa lipase was able to retain 94% of its activity after four hours at 50 °C and could be reused for up to 12 cycles [118]. The supports were selected due to their flexible and less ordered nature when compared to polyhydroxybutyrate. Byssus threads activated with 1,4-butanediol diglycidyl ether provided a suitable matrix for immobilizing urease, leading to enhanced pH stability and maintaining 50% of the activity of the enzyme under dry conditions [119]. In recent years, biocompatible mesoporous silica nanoparticles (MSNs) have gained attention as an environmentally sustainable support for biocatalysis. The use of these supports not only reduces production costs but also avoids ethical concerns. Due to their durability and effectiveness, MSNs have been applied in energy-related biocatalytic processes [120]. Table 1 presents the benefits, challenges, and solutions for overcoming the limitations of the adsorption technique.

**Table 1:** Common immobilization techniques, their advantages and disadvantages, and suggested approaches to overcome limitations.

Immobilization Technique	Advantages	Drawbacks	Approaches to Address the Limitations	Ref.
Adsorption	Prevention of proteolysis Full activity retention	Non-targeted adsorption The expense of affinity binding The activity is affected by a slight shift in the reaction conditions The leaching of enzymes	Using a blocking agent to reduce interactions that aren’t specific Specific pH for the charge difference between the silica support and the enzyme Pore size decrease following adsorption	[121,122,123]
Entrapment	Moderate preparation circumstances Prevents direct contact with the environment outside	Limited mobility on mass transfer Leakage is the result of fewer physical restraints	Exact pore size selection based on enzyme size Further covalent fusion	[124,125,126]
Encapsulation	Maintenance of enzymatic activity over prolonged periods Easy passage of small substrate molecules Large enzymes confined within the capsules	Difficulties in ensuring optimal diffusion of substrates and products Maintaining the structural integrity of the capsules under operational conditions	Development of capsules with improved stability, selectivity, and permeability Advances in materials science for better encapsulation materials and methods, such as 3D capsules	[126,127,128,129]
Covalent binding	Reduced limitations of mass transfer Improved storage and stability of reaction Stronger bonding	Specific binding site Denaturation of the enzyme’s active site Irreversible binding	Support and enzyme modification Specific binding site	[130,131,132]
Cross-linking	Aggregates may experience increased activity Recyclability, higher loading capacity, and total activity retention	The cross-linking matrix’s fragility Agents that precipitate conflict The pure enzyme is necessary for cross-linking enzyme crystals	The ideal aggregate size determined by the cross-linker-to-enzyme ratio Stabilizing components for the structure Using cross-linking enzyme crystals for enzymes that haven’t been fully purified	[130,133,134,135]

#### 2.1.2. Entrapment

Entrapment involves confining enzymes within gels or fibers using covalent or non-covalent bonds [136]. Effective entrapment has been realized with hybrid carriers made of alginate, gelatin, and calcium, which prevent the enzyme from leakage and offer increased mechanical stability [137]. The implementation of nanostructured materials in enzyme immobilization, such as pristine materials and electrospun nanofibers, which are produced through a method known as electrospinning, has significantly impacted the field. Mesoporous silica entrapment has recently emerged as a highly promising technology in fields such as biomedicine, fine chemistry, biosensors, and biofuels. This is largely due to the material’s unique properties, including a large surface area, uniform pore distribution, adjustable pore size, and high adsorption capacity. These features enable mesoporous silica to serve as an effective support material for various applications [138]. Lipase and magnetite entrapment of nanoparticles simultaneously within biomimetic silica has been shown to increase activity with various silane additives [139]. In the meantime, the selective binding and carrying properties of sol–gel matrices with supramolecular calixarene polymers have been used to entrap C. rugosa lipase [140,141]. In Table 1, entrapment’s advantages, disadvantages, and strategies to tackle its limitations are summarized.

#### 2.1.3. Encapsulation

The method of encapsulation immobilization entails the confinement of a variety of biomolecules within distinct polymeric structures [142]. This process shares similarities with entrapment, as both techniques permit enzymes and cells to exist freely within a solution while remaining in a controlled environment. Encapsulation aims to secure delicate enzymes and cellular solutions within small vesicles with porous barriers, preventing larger enzymes from exiting or entering the capsules, while smaller substrates or products can traverse the semi-permeable barrier with ease [127]. This method allows for the preservation of biological systems within a thin protective film, preventing direct environmental exposure that could negatively affect the performance of the biocatalysts, hence, enabling the prolonged activity of these biocatalysts [143]. Various supportive materials, such as cellulose nitrate and nylon, are employed in the production of microcapsules that range in size from 10 to 100 μm [144]. Furthermore, the process of ionotropic gelation of alginates and nanoporous silica-based sol–gel glasses has proven its efficacy in the field of enzyme encapsulation.

The simplicity of the encapsulation process distinguishes it, and advancements in material sciences have led to the improvement of this method, with benefits such as increased morphological stability, customizable physicochemical permeability, and reduced enzyme leakage [144]. The technique also offers the potential for co-immobilization, allowing for the possibility of immobilizing enzymes in any combination as required. Nevertheless, the method is not without its limitations. For example, issues related to diffusion can be significant, with the risk of membrane rupture if reaction products accumulate rapidly [128]. Table 1 provides a summary of the benefits, limitations, and strategies to overcome the challenges associated with the encapsulation method.

### 2.2. Chemical Techniques

Chemical methods involve the formation of strong covalent bonds between the enzyme and the support, leading to higher stability and reusability. Chemical techniques include covalent binding, cross-linking, and affinity immobilization. Covalent binding attaches enzymes to supports through covalent bonds formed with specific amino acids in the enzyme’s side chains [124]. Cross-linking forms covalent bonds between enzyme molecules using bifunctional or multifunctional agents. Affinity immobilization is a technique that utilizes the enzyme’s specific binding properties to support materials under different physiological conditions [104,145].

#### 2.2.1. Covalent Binding

Enzymes can be attached to supports through covalent binding, which relies on specific amino acids in the enzyme’s side chains, such as arginine, aspartic acid, and histidine. The effectiveness of this process is largely determined by the reactivity and efficiency of the functional groups present in the support, such as imidazole, indolyl, and phenolic hydroxyl [136]. Utilizing surfaces modified with peptides for enzyme immobilization leads to enhanced specific activity and stability of the enzymes, as well as the regulated orientation of the proteins [146]. Using CNBr-activated agarose and CNBr-activated sepharose, which have carbohydrate moieties and glutaraldehyde as a spacer arm, is one method for covalently attaching enzymes to supports. According to studies, this immobilization strategy has proven to give the linked enzymes thermal stability [117,147]. Through covalent enzyme attachment, silica gel carriers modified with silanization and SBA-15 supports with Si-F-lined cage-like pores created highly stable and hyperactive biocatalysts [148]. The enhanced half-life and thermal stability of enzymes have been achieved via covalently attaching them to various supports such as mesoporous silica and chitosan [138,147]. Covalently linking enzymes to electrospun nanofiber leads to improved residual activity as a result of greater surface area and porosity. The implementation of nanodiametric supports has revolutionized biocatalyst immobilization [149,150,151,152,153]. Alcohol dehydrogenase was covalently bound to attapulgite nanofibers (hydrated magnesium silicate) due to their thermal endurance and varying nanosizes [154]. Cross-linked enzyme aggregates have been developed by precipitating enzymes from aqueous solutions using organic solvents or ionic polymers [155]. The pharmaceutical industry has found covalent binding to magnetic nanoclusters to be useful in achieving varied orientations of immobilized enzymes. This approach has resulted in enhanced operational stability, durability, and reusability, making it a promising technique for enzyme immobilization [156]. One important function of cross-linking agents in enzyme immobilization is to maintain the enzymes’ structural and functional integrity. Glutaraldehyde is a commonly used bifunctional cross-linker that can form stable covalent bonds both within and between enzyme subunits, thereby preserving the enzyme’s activity and structure. It is also soluble in aqueous solvents, making it a convenient option for use in enzyme immobilization processes. Table 1 outlines the advantages, drawbacks, and approaches to address the limitations associated with covalent binding.

#### 2.2.2. Cross-Linking

Cross-linking is a method of immobilizing enzymes that do not require a support material and results in irreversible binding, preventing the enzyme from leaking into the substrate solution [145,157,158]. This immobilization technique, referred to as carrier-free immobilization, allows the enzymes to act as their carrier, thus resulting in a pure enzyme product and avoiding the drawbacks of using carriers [125,159]. The addition of carriers for enzyme immobilization may result in a decrease in activity, as the presence of non-catalytic components, referred to as ballast, can account for a significant proportion of the total mass, ranging from 90% to over 99%, ultimately leading to reduced space-time yields [155,159] and increased costs [155].

Cross-linking is a process of forming covalent bonds between enzyme molecules using bifunctional or multifunctional agents. One of the commonly used cross-linking agents is glutaraldehyde, owing to its affordability and large-scale availability [125,160]. For several decades, glutaraldehyde has been extensively utilized as a cross-linking agent to generate intermolecular cross-links between proteins, such as enzymes. The cross-linking of enzymes occurs through a reaction with free amino groups of lysine residues on neighboring enzyme molecules. This results in the formation of oligomers or polymers through both inter- and intramolecular aldol condensations, with the specific type of cross-linking dependent on the pH [155,161]. 

Cross-linked enzyme aggregates (CLEAs) are formed by precipitating enzymes with ammonium sulfate, acetone, or ethanol and then treating the aggregates with a cross-linking agent [125]. There are three methods for immobilizing enzymes, which are: (1) the blending of prepolymer and photosensitizer followed by gelling under near-UV radiation, (2) the freezing of enzyme-containing monomer solution into beads and subsequently polymerizing by gamma radiation, and (3) chemical polymerization via the combination of enzymes with acrylamide monomer and a cross-linking agent in a buffered aqueous solution. [162]. Lately, nanodiametric supports have induced significant advancements in biocatalyst immobilization [56,96,163,164]. The cross-linking immobilization of enzymes on electrospun nanofibers has been shown to improve residual activity, ascribed to the larger surface area and porosity of the substrate. CLEAs were employed to immobilize lysozyme on electrospun chitosan (CS) nanofibers, yielding a durable antibacterial material that can be used continuously [165]. Table 1 provides an overview of the merits, drawbacks, and methods to address the challenges related to the cross-linking technique.

#### 2.2.3. Affinity Immobilization

The affinity immobilization of enzymes involves utilizing their specific binding properties to support materials under different physiological conditions. There are two approaches to achieving this: first, by linking an affinity ligand specific to the target enzyme to the matrix, or second, by attaching the enzyme to a molecule that develops an affinity for the matrix [166]. The use of affinity adsorbents has not only been limited to the purification of enzymes but has also been extended to their simultaneous purification [167]. Sophisticated affinity matrices like chitosan-modified porous silica beads that are stable in alkali environments and multilayered concanavalin A attached to agarose are capable of immobilizing greater amounts of enzymes leading to better stability and efficiency [168,169]. The technique of bio affinity layering is an improvement over affinity immobilization, and it can significantly increase the capacity for enzyme binding and reuse. The non-covalent interactions, such as van der Waals forces, coulombic forces, and hydrogen bonding, among others, are utilized for this purpose [169,170].

## 3. Wastewater Treatment Using Enzymes Immobilized on Graphene Materials

### 3.1. The Synthesis of Graphene-Based Materials

Graphene sheets can be produced using various methods, such as mechanical and thermal abrasion, solvent extraction, and vapor deposition [171]. Chemical vapor deposition (CVD) is the most prevalent. CVD, a temperature-sensitive process, takes place in a reaction chamber where harmful oxygen molecules are distributed on the surface, and waste gases are removed [172]. Despite producing high-quality graphene sheets, CVD can generate hazardous byproducts. The process consists of two stages: pyrolysis of a reactant substance to form a carbonyl group on a substrate, followed by a heat-intensive procedure where fragmented carbon atoms assemble onto a substrate to create a single-layer framework [173]. Copper is a common substrate for producing high-quality graphene, as it bonds with carbon atoms and forms a single graphene layer on the surface. Using copper oxide between layers can facilitate the removal of a single graphene layer, while machining the copper substrate can lead to fewer defects in the final graphene product [174].

#### 3.1.1. Graphene Oxide

GO is commonly produced by oxidizing graphite oxide, which introduces numerous oxygen-containing organic compounds to the graphene layers’ interface. These functional groups promote layer separation and solubility in water [175,176]. Due to GO’s hydrophilicity, it can be subjected to ultrasonic waves, yielding a stable single graphene layer when dispersed in deionized water and other solvents. GO possesses remarkable characteristics, such as easy dissolution in various mediums, including water, ethanol, and different matrices. Its versatile nature, derived from the combination of electron-rich reactive oxygen and an electron-rich graphene framework, allows for extensive surface treatment and a wide range of applications. However, GO exhibits poor electrochemical properties and acts as an insulator in various phase separation solutions, while activated carbon remains permeable [177,178].

#### 3.1.2. Reduced Graphene Oxide

The three most common methods for converting GO to reduced graphene oxide (rGO) are chemical, thermal, and chemical treatment. Other techniques include hydrazine vapor treatment, and heat, light, and microwave reduction [179,180]. The reduction process is vital in rGO synthesis, determining the similarity between the rGO structure and GO precursors. While some commercial graphene nanoplatelet manufacturers supply an industrial-scale rGO equivalent, the scientific rGO used in nanoenabled products is distinct [181]. Chemical reduction is possible but often produces low yields and uses hazardous chemicals, resulting in rGO with low surface area and permeability compared to GO. Thermal reduction creates rGO with a large surface area and water volume like pure graphene, but causes structural damage due to the high carbon dioxide build-up [180,182]. Despite challenges in universality and viability, an electrochemical reduction is the most effective method, yielding rGO with properties like pristine graphene, a high carbon-to-oxygen ratio, and a resistance comparable to silver without generating harmful waste.

#### 3.1.3. Graphene Nanoplatelets

Graphene nanoplatelets (GNPs) can be produced through the nanoindentation fracture of graphene sheets, though this method yields a limited number of graphene flakes mixed with nanocrystalline layers. Hydraulic breakage, commonly employed in large-scale GNP synthesis, is subjected to chemical reduction to obtain the final GNP product [183,184]. Plasma abrasion is another technique for generating substantial GNPs, with the advantage of creating and coating GNPs in a simple, dry step to enhance diffraction in the host polymer. The material is purified using a vacuum in an RF or Microwave Plasma Reactor to eliminate impurities and residual pollutants from plasma machining processes [185]. With a range of accessible organic compounds and reduced costs for raw materials, capital equipment, plasma purification, and functionalization, GNPs could eventually become more cost-effective than (CNTs) on a large scale, promoting increased benefits for early investors [183,184].

### 3.2. Enzyme Immobilization on Graphene-Based Materials

Graphene and GO have garnered considerable interest as enzyme support materials among carbon-based substances, thanks to distinctive characteristics such as their biodegradability, two-dimensional structure, extensive surface area, pore volume, and excellent chemical and thermal stability [186,187]. Different chemical groups, like hydroxyl (–OH), carboxylic (COOH), and epoxide groups, can form strong enzyme-matrix interactions without the need for coupling agents or modifying the graphene surface [188]. Consequently, enzymes like peroxidases [93] and lipases [189] can be immobilized on GO surfaces using adsorption, covalent binding, or entrapment [190]. Graphene-based materials might even boost enzyme biocatalytic activity and possess antioxidant properties, aiding in the elimination of free radicals (e.g., hydroxyl or dithiocyanate) from reaction mixtures and enhancing enzyme protection against inactivation [191]. Due to its water solubility and extensive surface area with oxygen functionalities, GO has emerged as an especially promising material for immobilizing proteins and enzymes, negating the need for the pre-modification of the surface [93,192]. Nevertheless, despite the immense potential of GO, research on its effects on the catalytic properties of immobilized enzymes is still limited, and the available results are challenging to compare due to differences in the enzymes and methods of immobilization. For instance, Hernandez-Cancel et al. [193] performed the first investigation on the immobilization of bilirubin oxidase on GO sheets to understand the influence of chemical glycosylation and immobilization on the enzyme’s catalytic properties. They revealed that glycosylation, which is an example of enzyme immobilization on graphene materials for pollutant removal, decreased its catalytic properties while increasing thermal stability.

An example of the use of immobilized enzymes is the employment of HRP immobilized on the nanoparticles of rGO that have been treated with glutaraldehyde, which has shown significant improvements in the enzyme’s kinetic parameters and the enzyme’s ability to be reused [194]. Likewise, d-psicose 3-epimerase (DPEase) immobilized on unmodified GO displayed increased biocatalytic conversion efficiency and superior thermal stability [195]. Although some studies in the literature have reported a decline in biocatalytic activity, recent investigations suggest that employing nanoparticles as enzyme carriers can preserve or even boost the efficiency of immobilized enzymes [196,197]. For example, lipase immobilized on GO showed a 55% increase in hydrolytic activity [198], and trypsin’s activity for casein digestion was enhanced when bound to PEG-coated GO nanosheets [199]. Wei and Ge [200] examined the influence of GO on the conformation and activity of immobilized catalase, discovering that changes in protein structure induced by GO led to a reduction in catalytic ability, with these alterations being reliant on the carrier concentration and duration of the interaction. According to Zhang et al. [201], the immobilization of HRP onto a nanostructure GO support has been shown to considerably improve its thermal stability. However, this positive effect diminishes at high temperatures. A study by Chang et al. [202] reported that the immobilized HRP’s activity on a nanocomposite of superparamagnetic Fe_3_O_4_/GO decreased to 40% of its initial activity when subjected to a temperature of 70 °C, which is 40 °C above the optimum temperature.

### 3.3. The Remediation of Dyes by Enzymes Immobilized on Graphene-Based Materials

Exploring the potential of enzyme immobilization on graphene materials for the removal of organic dyes has become a focal point in recent research, contributing to the development of efficient treatment methods. In a study by Xu et al. [203], bi-functional hemin–graphene nanosheets were created and effectively used for the adsorption of dye pollutants from an aqueous solution. Bovine serum albumin (BSA) was used by Wang et al. [204] to develop a green synthesis method for bi-functional hemin–graphene nanosheets, which were efficiently utilized for the adsorption of dye contaminants from an aqueous solution. In these bi-functional systems, GO served to adsorb the dye while simultaneously immobilizing the enzymes catalytically degrading it [204]. This approach has informed the field of catalytic applications, where the integration of GO with metal nanoparticles has been of significant interest to reduce dye pollution catalytically utilizing reducing agents like NaBH4 and hydrazine hydrate [205,206].

In a related study, Patila et al. [207] conducted the multipoint covalent immobilization of laccase onto functionalized GO (fGO) and found that the catalytic activity of the resulting nanoassemblies was dependent on the number of GO-laccase layers. The authors found that, compared to free enzymes, the immobilized laccase exhibited 4.7 times greater thermal stability at 60 °C. Furthermore, it showed a 10% improvement in decolorizing pina cyanol chloride, an industrial dye. The immobilized enzyme also showed excellent reusability, retaining almost complete decolorization activity after five reaction cycles. In a similar vein, Kashefi et al. [208] developed a large-scale and straightforward method for covalently immobilizing laccase onto GO nanosheets. The synthesized immobilized enzymes and nanomaterials were characterized and confirmed to be successful through the decolorization experiments of Acid Blue 92 and Direct Red 23 dyes. The nanobiocatalyst had a reusability rate of over 75% after six cycles, indicating the effectiveness of the immobilization process. The work of Ariaeenejad et al. [209] focuses on the improved efficacy of dye removal, such as Methylene Blue, from water through the use of a recently developed immobilized enzyme on modified magnetic graphene oxide (GO), which has demonstrated dual functionality. PersiManXyn1, a model enzyme, was covalently attached to an amine-functionalized GO nanocarrier. The immobilized enzyme exhibited excellent thermostability, retaining more than 35% of its activity at a high temperature of 90 °C, while the free enzyme maintained only 5% of its maximum activity. Furthermore, even after a storage period of four weeks, the immobilized enzyme retained 54% of its initial activity, while the free enzyme was deactivated. The immobilized PersiManXyn1 effectively removed Methylene Blue from water using two distinct approaches. In contrast to the negligible catalytic ability of the pristine nanocarrier and free enzyme, the immobilized PersiManXyn1 rapidly reduced concentrated Methylene Blue solutions within 150 s and exhibited excellent reusability (94% dye removal after the 15th cycle).

Expanding on these findings, Vineh et al. [210] covalently immobilized HRP onto functionalized reduced graphene oxide-SiO_2_. The immobilized HRP showed a 100% removal efficiency for a phenol concentration of 2500 mg/L, whereas the free HRP achieved a removal efficiency of only 50%. In the case of immobilized HRP, most dyes were decolorized completely with 100% efficiency. Meanwhile, Ali et al. developed a simple adsorption mechanism to immobilize GP onto a novel nanocomposite, PCeGONC. The activity of GP was enhanced through the immobilization process, with a recuperation rate of 128% of the original activity. This immobilized GP showed improved efficiency in decolorizing Reactive Blue 4 dye compared to free GP, reaching a decolorization efficiency of 99% within a 3 h stirred batch treatment. Additionally, the immobilized GP demonstrated greater operational stability, maintaining roughly 72% of its initial activity even after 10 separate rounds of dye decolorization in a batch method. Table 2 provides further information on the use of enzyme immobilization on GO for dye removal.

**Table 2:** Enzyme immobilization on graphene-based materials, CNTs, and MOFs supports for dyes and phenolic compound remediation with the reported optimum condition and removal %.

Dye						
Media	Enzyme	Techniques	Optimum Condition	Pollutant	Removal (%)	Ref.
GO nanosheets	Genetically Modified Aspergillus Laccase	Covalent binding	60 min, pH 5, 45 °C	Acid Blue 92 Direct Red 23	75 75	[208]
Polypyrrole-cellulose- GO nanocomposite	Peroxidase	Non-covalent binding	100 min, pH 4, 40 °C	Reactive Blue 4	99%	[211]
GO	Porcine pancreas lipase	Adsorption	240 min, pH 8, 40 °C	Azo dyes	89.47	[212]
GO	Laccase	Adsorption	24 h, pH 5, 50 °C	Crystal Violet Reactive Brilliant Blue Methyl Orange Reactive Brilliant Blue 14	Better than 40%	[203]
GO	Manganese Peroxidase	Covalent binding	5 h, pH 5, 35 °C	Azo dye Triphenylmethane dye Anthraquinone dye	Better than simple enzyme	[213]
GO	Laccase	Cross-linking	60 min, pH 3, 45 °C	Direct Red 23	91	[214]
CNTs/GO	Laccase from trametes versicolor	Adsorption	20 °C	Methylene Blue	80	[215]
CNTs	Laccase from trametes versicolor	Cross-linking	24 h, pH 5, 25 °C	Methylene Blue Orange II dye	96 74	[216]
CNTs	Laccase from trametes versicolor	Adsorption	3 h, pH 7, 35 °C	Congo Red	96	[94]
CNTs	Ganoderma lucidum’s LiP	Covalent binding	24 h, pH 3.5, 25 °C	Remazol Brilliant Blue R	78	[217]
Fe_3_O_4_-MWCNTs@SiO2	Laccase from Trametes versicolor	Covalent binding	3.5 h, pH 3, 60 °C	Acid Red 88 Reactive Black 5 Eriochrome Black T	98, 99, 66	[218]
MWCNTs	Laccase from myceliophthora Thermophile	Covalent binding	24 h, pH 5, 25 °C	Reactive Black 5	84	[219]
Cu-PABA (MOFs)	Laccase	Encapsulation	6 h, pH 4.5, 40 °C	Direct Red 31	92	[220]
Cu-MOFs Co-MOFs Cu-MOFs Co-MOFs	Laccase	Encapsulation	1 h, pH 4.5, 50 °C 1 h, pH 5, 50 °C 1 h, pH 4.5, 50 °C 1 h, pH 5, 50 °C	Reactive Blue 171 Reactive Blue 171 Reactive Blue 198 Reactive Blue 198	89 88 39 77	[221]
Fe-BTC/NiFe_2_O_4_ (MOFs)	Laccase	Coprecipitation	1 h, pH 3, 22 °C	Methylene blue	100	[222]
Fe_3_O_4_@ZIF-8 (MOFs)	Laccase	Coprecipitation	30 min, pH 4.5, 70 °C	Crystal Violet Methylene Blue	93 91	[223]
NH_2_-MIL88 (Fe) (MOFs)	Laccase	Cross-linking	2 h, 30 °C	Remazol Brilliant Blue R	92	[224]
Fe_3_O_4_@ZIF-8 (MOFs)	Laccase	Coprecipitation	15 min, pH 7, 40 °C	Indigo Carmine	100	[225]
ZIF-8 (MOFs)	Laccase	Covalent binding	2 h, pH 3, 40 °C	Acid Blue 92	90	[226]
Fe_3_O_4_-NH_2_@MIL-101 (MOFs)	Laccase	Covalent binding and adsorption	2 h, pH 3, 25 °C	Alizarin Red S Reactive Black 5	100 81	[227]
Phenolic compound						
GO	HRP	Covalent binding	pH 5, 40 °C	Phenol	100	[194]
Nanostructure GO	HRP	Adsorption	30 min, pH 6	3-aminophenol Catechol 2-methoxy phenol Phenol 4-methoxy phenol 2,4-dimetheoxyphenol 2-CP	87.6 72.7 68 64 69 34.4 20.4	[201]
GO/Fe_3_O_4_	HRP	Covalent binding	3 h, pH 6, 25 °C	Phenol 2,4-DCP	70 100	[228]
Fe_3_O_4_/GO	HRP	Covalent binding	2 h, pH 6.4, 25 °C	2-CP 4-CP 2,4-DCP	23 44 83	[202]
rGO	Ochrobactrum sp. FJ1	Adsorption	10 days, pH 7, 25 °C	BPA	64.6	[229]
Functionalized MWCNTs	Laccase	Covalent binding	60 min, pH 5.6, 23 °C	Phenol Resorcinol 4-Methoxyphenol 4-CP	90 90 100 45	[230]
MWCNTs	Laccase	Cross-linking	300 min, pH 5, 35–45 °C	BPA	90	[231]
PAN-MIL-101 (Cr) (MOFs)	Laccase	Electrostatic adsorption	5 h, pH 5, 23 °C	BPA	92	[232]
NH_2_-MIL-53(Al) (MOFs)	Laccase	Non-covalent immobilization	0.5 h, pH 4.5, 21 °C	BPA	99	[233]
HKUST-1 (MOFs)	Laccase	Encapsulation	4 h, pH 6.5, 40 °C	BPA	98.2	[234]
Cu_3_(BTC)_2_ @P1 (MOFs)	Laccase	Encapsulation	12 h, pH 5, 40 °C	BPA	99.6	[235]
Cu-PABA (MOFs)	Laccase	Coprecipitation	12 h, pH 5.5, 35 °C	BPA	84.7	[95]
Graphene aerogel-Zr-MOFs	Laccase	Adsorption	24 h, pH 4, 40 °C	Hydroquinone	79	[236]
BC/c-MWCNTs/ZIF-90 (MOFs)	Laccase	Encapsulation	2 h, pH 4, 50 °C	Catechol	93.4	[237]
Fe_3_O_4_-NH_2_@MIL-101(Cr) (MOFs)	Laccase	Adsorption and covalent binding	2 h, pH 4, 25 °C	2,4-DCP	87	[238]
Fe_3_O_4_-NH_2_@MIL-100(Fe) (MOFs)	Laccase	Adsorption and covalent binding	200 min, pH 5, 50 °C	Nonylphenol polyethoxylated Octylphenol polyethoxylated	98.16 100	[239]

### 3.4. The Remediation of Phenols by Enzymes Immobilized on Graphene-Based Materials

Investigations into the use of enzyme immobilization on graphene materials for phenolic compound removal have demonstrated promising results, offering innovative solutions for environmental remediation. The use of GO as a matrix for immobilizing enzymes has been reported by Zhang et al. [93,201]. The authors of the study employed a cross-linking agent-free method to immobilize HRP and lysozyme onto GO sheets. This involved incubating the GO sheet in a phosphate buffer solution that contained the enzyme molecules. The concentration of the buffer solution in which the HRP was initiated was observed to affect the loading density of HRP on GO. AFM imaging allowed for the visualization of the immobilized enzymes, which can be difficult to achieve with other nanoscale solid substrates. The dimensions of the immobilized enzymes were estimated to be approximately 140 × 140 × 15 Å, which is similar in size to free HRP (30 × 60 × 75 Å). According to the authors, electrostatic interactions between enzyme molecules and negatively charged GO sheets (within a pH between 4 and 11) were the primary driving force behind the immobilization of HRP and lysozyme on GO. The thermal stability and pH range of the immobilized enzymes on GO were enhanced compared to their free counterparts. In addition, the immobilized enzymes showed high efficiency in removing various phenolic compounds commonly present in industrial wastewater, such as 2,4-dimetheoxyphenol and 2-chlorophenol (2-CP). These results showed that GO has exceptional potential as a solid substrate for enzyme immobilization. 

In a study by Wu et al. [240], a composite comprising graphene quantum dots and Fe_3_O_4_ nanoparticles was synthesized and tested for its ability to eliminate phenolic compounds in synthetic wastewater. The authors observed that the composite displayed higher removal efficiency than HRP, indicating its potential as a viable option for the removal of phenolic compounds from wastewater. In a study conducted by Chang et al. [202], Magnetic Fe_3_O_4_ nanoparticles were utilized as a support for the immobilization of HRP onto GO sheets, which were then used for the removal of 2-CP, 2,4-dichlorophenol (2,4-DCP) and 4-chlorophenol (4-CP) from contaminated water. The chlorophenol removal efficiency was impacted by the varying numbers and positions of electron-withdrawing substituents, following the order of 2,4-DCP > 4-CP > 2-CP. Gas chromatography mass spectrometry was employed to analyze the oxidation products generated during chlorophenol degradation. The NPs were retrieved using an external magnetic field, and the immobilized HRP retained 66% of its activity after four consecutive uses. These findings suggest that the immobilized enzyme is effective in treating hazardous phenolic compounds present in wastewater. Table 2 shows additional research on the application of enzyme immobilization on GO for the removal of phenolic compounds.

## 4. Wastewater Treatment Using Enzymes Immobilized on CNTs

### 4.1. Synthesis of CNTs

**Figure 1:**
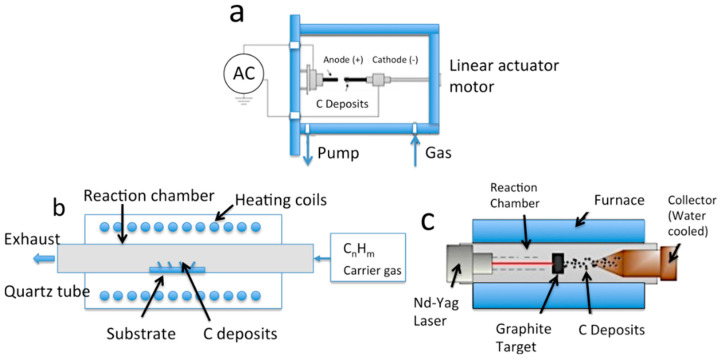
The diagram illustrates the various techniques employed for CNTs synthesis, including arc discharge (**a**), chemical vapor deposition (**b**), and laser ablation (**c**) [245].

Various techniques can be used to produce CNTs. These include the chemical vapor deposition (CVD) method, arc discharge method, laser ablation method, mechano-thermal synthesis method, flame synthesis method, and electrolysis method; some of the common methods demonstrated in Figure 1 [241]. The production of CNTs involves several techniques, including the arc discharge method and laser ablation method. In the method of arc discharge, two graphite electrodes are subjected to direct current arc voltage in an inert gas atmosphere, producing CNTs that are collected at the graphite cathode. The use of a pure graphite anode typically results in multi-walled carbon nanotubes (MWCNTs), while metal-doped graphite anodes generate single-walled carbon nanotubes (SWCNTs). Alternatively, the laser ablation method involves the use of a high-energy pulsed laser system to heat graphite samples in a high-temperature reactor, with the resulting CNTs transferred to a cooled collector using high-pressure inert gases [242]. In contrast to other methods of producing CNTs, CVD involves the continuous flow of hydrocarbon gases over a catalyst at high temperatures to create CNTs [243]. The mechano-thermal synthesis method has been investigated by researchers as an alternative to laser methods for producing CNTs. This process involves milling graphite flakes and then thermally annealing the resulting nanopowder at high temperatures to create the CNTs. The flame synthesis method, which requires no complex installations, utilizes a premixed flame consisting of fuel and an oxidizing agent directly applied to catalysts for CNTs production [243]. Finally, the electrolysis method is like the arc discharge method but with a graphite electrode immersed in solution to synthesize CNTs [244].

The chemical vapor deposition (CVD) technique is widely regarded as the most promising method for producing CNTs on a larger scale and with greater process adaptability. This is attributed to its economic benefits, reduced energy use, and more straightforward operation in comparison to alternative methods like arc discharge, laser ablation, mechano-thermal, and electrolysis. These other approaches depend exclusively on raw graphite materials, which constrains their ability to utilize various carbon precursors and scale up. Recent studies have demonstrated the potential to generate CNTs from plastic-derived pyrolytic gas using CVD, emphasizing the method’s versatility and potential for industrial-scale production. Moreover, the flame synthesis method generally underachieves in comparison to CVD, as it frequently leads to the creation of soot nanoparticles. This is likely due to the presence of OH radicals that erode CNTs in a continuous O_2_ supply environment, as well as unstable reactor conditions caused by uneven temperature profiles in substrates generated by flames [241].

### 4.2. Enzyme Immobilization on CNTs

CNTs are often utilized as carriers for biological applications owing to their robust mechanical properties and high chemical and thermal stability. Oliveira et al. [217] employed CNTs as a biological carrier for LiP to degrade Remazol Brilliant Blue R dye. The use of CNTs as a support resulted in immobilized enzymes with higher specific activity and reusability compared to free enzymes, which implies their potential in industrial applications for dye removal [217]. Xu et al. [246] attached the laccase enzymes to PVA/chitosan/MWCNTs, which were electrospun onto aluminum foil and activated with glutaraldehyde before enzyme immobilization [246]. These PVA/chitosan/MWCNTs-immobilized laccase membranes exhibited an enhanced enzyme loading capacity and activity retention compared to those without MWCNTs, achieving 100% diclofenac removal versus 84.9% removal with laccase PVA/chitosan [246]. CNTs were utilized as a biological carrier to immobilize LiP and facilitate the enzymatic degradation of Remazol Brilliant Blue R dye. The immobilized enzymes showed increased specific activity and reusability when compared to their free counterparts, indicating their potential as an effective tool for industrial dye removal applications [246].

CNTs have proven to be an effective support for immobilizing various enzymes used for wastewater treatment, such as Lip, laccase, and HRP. In their research, Oliveira et al. [217] demonstrated the potential for using CNT-immobilized LiP to degrade dyes. The immobilized enzyme showed improved catalytic efficiency and stability in comparison to the free enzyme. In addition, Chen et al. [247] introduced an innovative technique to immobilize laccase on magnetic GO to remove the dye. Their findings revealed that the laccase immobilized on magnetic nanoparticles demonstrated enhanced thermal and pH stability, elevated enzyme activity, and exceptional reusability compared to the free enzyme. Moreover, the catalytic efficiency of laccase immobilized on different carbon nanomaterials, including C60, MWCNTs, O-MWCNTs, and GO for phenolic compounds removal, was studied by Pang et al. [248]. Their findings revealed that, while the enzyme loading increased, the reaction rate decreased as compared to free enzymes.

### 4.3. The Remediation of Dyes by Enzymes Immobilized on CNTs

The use of enzyme immobilization on CNTs for removing organic dyes has gained considerable interest as an innovative solution for tackling pollution issues in the scientific community. A promising method entails treating textile-dye-contaminated wastewater with fungal extracts containing LiP. Oliveira et al. [217] fermented Jatropha curcas seed cake using Pleurotus ostreatus (PLO9) and Ganoderma lucidum (GRM117) to generate enzymatic extracts. These extracts were subsequently immobilized on CNTs, leading to enhanced LiP-specific activity compared to the free enzyme. The immobilized extracts exhibited higher Vmax and lower Km values than the free enzyme and demonstrated efficient reusability in dye decolorization, making them a promising biocatalyst for the process. Expanding upon this research, Lia et al. [177] created a biocatalyst by immobilizing laccase on cross-linked polymethacrylate (PMMA)/(CNTs) fabricated through miniemulsion polymerization and activated with glutaraldehyde (GA). The immobilized laccase displayed high reusability, maintaining around 50% of its initial activity after 10 batches. With exceptional storage capacity, stability across a wide range of working temperatures, and optimal pH stability, the immobilized laccase effectively decolorized both Methylene Blue and Orange II, achieving decolorization rates of up to 96% and 74%, respectively. The decolorization rate declined by approximately 10% after 10 consecutive cycles, making this approach a promising option for eliminating azo dyes from wastewater samples [216].

In a related study, Zhang et al. [94] employed laccase-immobilized CNTs to improve the removal of dyes from wastewater samples. The laccase–CNT nanocomposites surpassed traditional techniques in terms of pollutant elimination efficiency and catalytic degradation. The addition of the laccase enzyme averted CNT agglomeration and expanded the space between them, promoting more effective adsorption sites and yielding a removal rate of over 90% after 100 min of operation. The laccase–CNTs achieved an enhanced dye removal capacity of more than 96%, attributable to the even distribution of dye within the CNTs, which improved the mass transfer efficiency of pollutants. The combination of adsorption and catalytic biochemical degradation also contributed to greater particle diffusion in laccase–CNTs. In a complementary study, Othman et al. [219] attached Myceliophthora thermophila laccase to functionalized MWCNTs, forming a bio-barrier with impressive operational stability and resilience to temperature and pH changes. The immobilized laccase demonstrated strong operational stability, preserving over 95% of its starting activity after ten reaction cycles. For example, when exposed to 20% acetone (*v*/*v*) for 6 h, the stability of free and immobilized laccase was 21% and 49%, respectively. The researchers employed the immobilized laccase to decolorize Reactive Black 5 dye, reaching a 68.09% decolorization rate after 6 h and 84.26% after 24 h. The procedure was refined by incorporating 1-hydroxy benzotriazole as a mediator and sustaining a pH of 5.0. Table 2 presents a comprehensive overview of the research on enzyme immobilization on CNTs for dye removal.

### 4.4. The Remediation of Phenols by Enzymes Immobilized on CNTs

The removal process of phenolic compounds using nanotubes has gained attention in environmental research, offering the potential for significant contributions to sustainable practices. Dai et al. [231] developed MWCNTs integrated with laccase electrospun fibrous membranes (LCEFM) for bisphenol removal in water [231]. The researchers employed an in situ electrospinning technique to produce MWCNTs-LCEFM nanofibers. To prepare the polymer solution, PDLLA was mixed with laccase initially to stabilize the enzyme before being incorporated with MWCNTs. The study showed that the MWCNTs-LCEFM nanofibers contained active laccase, and the enzyme was uniformly distributed within the fibers, as observed from the pore edges. The addition of 1.5 wt% PDLLA to the MWCNTs and laccase mixture resulted in an approximately threefold improvement in the specific surface area and mechanical tensile strength of the MWCNTs-LCEFM compared to LCEFM. The MWCNTs-LCEFM nanofibers displayed an improved catalytic activity of 89.9% and superior storage and operational capabilities compared to LCEFM. The modified nanofiber membrane was able to remove triclosan, bisphenol A (BPA), and 2,4-DCP from wastewater, with adsorption efficiency for BPA and 2,4-DCP increasing by 50%. MWCNTs-LCEFM achieved degradation rates of 99.7% ± 0.02%, 95.5% ± 0.46%, and 92.6% ± 0.74% for triclosan, BPA, and 2,4-DCP, respectively. Additionally, MWCNTs-LCEFM demonstrated practical potential for treating emerging organic pollutants in wastewater, as degradation efficiencies of 90.5% ± 1.1%, 85.6% ± 1.5%, and 81.7% ± 1.9% were recorded for BPA, triclosan, and 2,4-DCP, respectively [231]. 

In a study by Costa et al. [230], multi-walled carbon nanotubes (MWCNTs) that had been chemically functionalized were employed as carriers for the immobilization of laccase. The MWCNTs were altered using various techniques and methods in combination. The most favorable balance between recovered activity and immobilization efficiency was achieved using MWCNTs functionalized with 0.3 M HNO_3_ and treated with N-hydroxysuccinimide and N-ethyl-N-(3-(dimethylamino)propyl) carbodiimide hydrochloride. The thermal stability of this catalyst was found to be exceptional at temperatures of 50 and 60 °C. Based on the results of the reusability test, the laccase activity remained above 65% of its initial value after five consecutive cycles of reuse. The immobilized enzyme’s biocatalytic performance was assessed for the degradation of a phenolic compound mixture in water consisting of resorcinol, phenol, 4-chlorophenol, 4-chlorophenol, and 4-methoxyphenol, with removal rates of 90, 90, 45, and 100%, respectively. For additional research on the application of enzyme immobilization on CNTs for the removal of phenolics, Table 2 can be referenced.

## 5. Wastewater Treatment Using Enzymes Immobilized on MOFs

### 5.1. Synthesis of MOFs

MOFs are a type of framework structure composed of metal ions or clusters coordinated with organic ligands [124]. The synthesis of these frameworks involves the combination of metals and organic skeletons, resulting in highly porous structures with tunable and ultrahigh porosity, structural flexibility, chemical and thermal stability, large surface areas, and multiple functional sites [225,249,250]. In addition, MOFs can be modified by synthetic methods that enable the introduction of specific functional groups into their organic bridging ligands, allowing for the customization of the framework to meet specific requirements or applications [251]. The synthesis of MOFs involves dissolving metal precursors and organic linkers in solvents and then placing them in a sealed reaction vessel, leading to the self-assembly of MOFs crystals. This is usually completed through a solvothermal method, which is carried out at a temperature below 220 °C, and it can take several weeks for the crystals to form. Common solvents used in this process include ethanol, methanol, acetonitrile, N, N-dimethylformamide (DMF), and N, N-dimethylformamide (DEF) [252,253,254].

**Figure 2:**
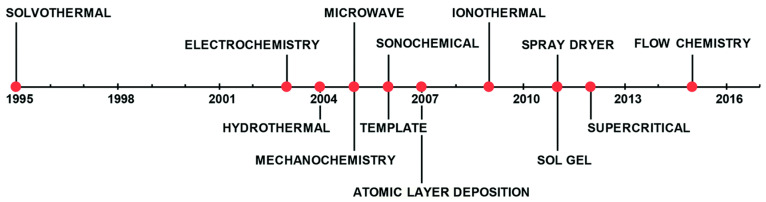
A chronology of the prevalent patented methods for synthesizing MOFs [235].

**Figure 3:**
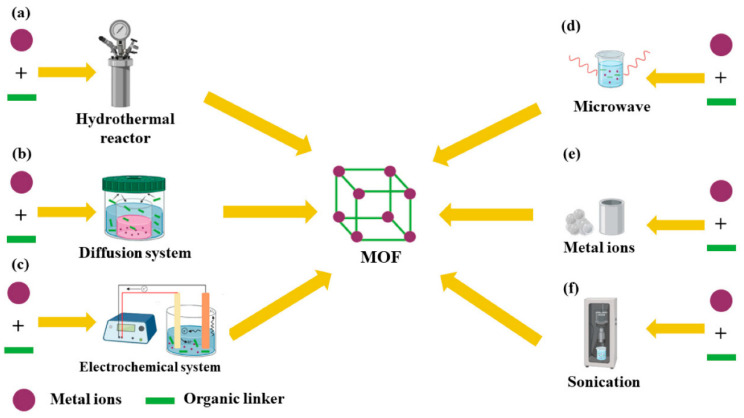
The diagram depicts various techniques for MOFs synthesizing, including solvothermal and hydrothermal (**a**), diffusion (**b**), electrochemical (**c**), microwave-assisted (**d**), mechanochemical (**e**), and sonochemistry (**f**) [255].

After more than twenty years of research and development, significant progress has been achieved in MOFs synthesis. Additional synthesis methods have been reported, including electrochemical, microwave-assisted, mechanochemical, and microfluidic synthesis methods, among others [235]. The timeline in Figure 2 below outlines the progression of the most commonly employed MOFs synthesis methods over time, while Figure 3 demonstrates some of the common methods to synthesize MOFs.

Numerous synthesis approaches have expanded the number of MOFs structures synthesized since their initial discovery. The principle of “structure dictates function” is evident in this field [256]. The control and customization of the morphology, size, and chemical functionalization of MOFs crystals are crucial for achieving specific properties and optimal performance in resulting MOFs materials. Achieving this requires advanced synthesis strategies based on an understanding of the crystallization mechanisms that occur during synthesis. Several modulated synthesis methods have been developed to achieve control over MOFs crystal morphology and size, as well as doping to create hybrid MOFs crystals.

### 5.2. Enzyme Immobilization on MOFs

MOFs represent a promising category of materials with unique properties suitable for a range of applications, including catalysis, storage, separation, purification, and water remediation [257,258,259,260,261,262]. The immobilization of laccase on MOFs has been achieved using a variety of metals and metal oxides, including iron [263], copper [249], zeolite [225], and zirconium [264]. MOFs, unlike traditional inorganic materials, allow for precise control over composition, morphology, pore properties, and function by carefully selecting construction units and incorporating intelligent functionalities. The ability to control the properties of MOFs is crucial for improving their performance in specific applications [265,266,267]. The catalytic activity of MOFs is generated from uncoordinated metal centers or functional groups linked to the structure’s ligands [268]. In addition, MOFs can also serve as support for catalysts, including nanoparticles, metal complexes, or biomolecules, by either housing them within the MOFs cage or anchoring them to the MOFs surface. This approach can provide size-selective catalyst support and stabilize active catalysts [269,270].

**Figure 4:**
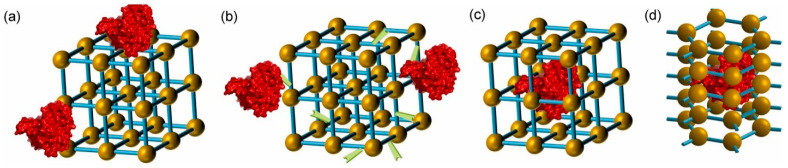
Typical methods used to synthesize enzyme-MOFs biocomposites include physical adsorption (**a**), covalent binding (**b**), encapsulation (**c**), and pore trapping (**d**) [254].

MOFs have become a promising candidate for enzyme immobilization due to their numerous advantages, such as large specific surface area, high pore volume, customizable porosity, high thermal and chemical stability, and adjustable mechanical stability. These distinctive properties of MOFs enable them to accommodate a higher loading of enzymes compared to conventional carrier materials, and to stabilize the conformational structure of enzymes, thereby improving their stability [271,272]. MOFs can modulate enzyme properties by enabling different functionalizations, sizes, morphologies, and electrostatic potentials [273,274]. The ability of MOFs to adapt to extreme conditions, including high temperatures, acidic or alkaline media, and organic solvents, is essential for ensuring the efficient stabilization and activity of enzymes. As shown in Figure 4, there are four common strategies used for the preparation of MOFs-immobilized enzymes: encapsulation, physical adsorption, covalent binding, and pore trapping with pre-synthesized MOFs. These approaches offer flexibility and allow for the effective immobilization of enzymes, ultimately leading to enhanced stability and catalytic activity [275]. Enzyme stability can be enhanced in harsh environments, including high temperatures, organic solvents, and extreme pH levels, by encapsulating enzymes within porous materials such as porous nanoparticles or reversible micelles [129]. The potential for synergistic catalysis between MOFs and enzymes holds great promise for industrial applications [274,276].

Cui et al. [277] proposed a technique for creating a durable and reusable MOFs-enzyme composite by incorporating catalase and ZIF-8 nanocrystals into multiple layers of mesoporous silica through encapsulation. The composite exhibited a high activity recovery rate of up to 81%, where the silica layer served as a shield against chemical and biological degradation. In addition, the composite demonstrated exceptional stability even in harsh conditions such as low pH and against proteolytic agents, retaining 50% of its initial activity even after ten cycles of use [277]. In a separate study by Ladole et al. [223], the researchers produced magnetic MOFs (MMOFs) containing magnetic nanoparticles and peroxidase. The researchers utilized nanocrystalline NH_2_-MIL-53 (Al) to encapsulate laccase and develop a biocatalyst with desirable properties such as high thermal stability, excellent residual activity even after several reaction cycles, and long-term storage stability. Entrapping laccase in NH_2_-MIL-53 (Al) allowed for high enzyme loading and permanent retention, resulting in a biocatalyst with an average diameter of 100 nm. The biocatalyst effectively eliminated BPA from water, with complete removal achieved within three minutes [233]. 

### 5.3. The Remediation of Dyes by Enzymes Immobilized on MOFs

Enzymes, especially laccases, have shown success in eliminating dyes from wastewater when immobilized on MOFs [278]. MOFs can contribute to the dye removal process via adsorption [223,224,279]. Certain MOFs-based supports display fast adsorption processes, accounting for a substantial part of dye elimination in the early stage. As the support reaches saturation, dye removal primarily takes place through laccase-catalyzed degradation. For example, the adsorption–degradation process was followed by the laccase included in MIL-68(Al)/PVA’s degradation of Alizarin Green (AG). This composite MOFs rapidly adsorbed AG, attaining a 65.32% removal rate within 2 h. The removal efficiency rose to 95.86% with additional laccase degradation. However, as the support was reused more often, the adsorption impact decreased [254].

The continuous degradation of dyes can be accomplished through the utilization of bioreactors containing immobilized laccase as an alternative to the conventional batch method. Ladole et al. [223] demonstrated this by introducing laccase@MMOFs into a glass column for the continuous degradation of Crystal Violet and Methylene Blue [223]. By employing immobilized laccase in a bioreactor, the mass transfer rate and interaction between dyes and the enzyme were improved, leading to a slightly enhanced degradation efficiency in comparison to the traditional batch method. The application of MOFs-based immobilized laccase showed a higher removal efficiency due to the combined effect of adsorption and biodegradation, surpassing that of free enzymes [254].

MOFs-based support can enhance the catalytic properties of laccase, leading to increased dye decolorization efficiency. Yang et al. [222] created magnetic micromotors (Fe-BTC/NiFe_2_O_4_-MT) with peroxidase activity and utilized them for the immobilization of laccase. When tested in a solution containing 3% H_2_O_2_, the laccase@Fe-BTC/NiFe_2_O_4_-MT was able to completely decolorize MB within 20 min, whereas the free enzyme was only able to decolorize 15% of MB. The Fe-BTC/NiFe_2_O_4_-MT nanoenzyme played a crucial part in breaking down MB, while the immobilized laccase oxidized MB and its degradation byproducts. The micromotor’s propulsion, driven by oxygen bubbles produced from H_2_O_2_ decomposition, expanded the reaction’s contact area and hindered product buildup. The generated oxygen also took part in laccase catalysis, facilitating the reaction [222].

Laccases show promise not just in the effluent of dye treatment but in textile bleaching as well. Bioscouring, a technique that employs enzymes instead of chemicals, can enhance product quality and decrease resource usage [280]. To improve ZIF-8’s bleaching ability, Madurella mycetomatis laccase (MmLac) was immobilized in silica-modified ZIF-8. Compared to control tests utilizing simple chemical agents, free MmLac and silica@MmLac/ZIF-8 demonstrated improved bleaching efficiency [281,282]. Silica@MmLac/ZIF-8 exhibited a 2.3-fold increase in bleaching efficiency compared to the free enzyme, indicating its potential application in textile bleaching. As the laccase-MOFs composite exhibits high efficacy and low environmental impact, it can be considered a promising alternative for the textile industry [283]. Table 2 contains more studies on the utilization of enzyme immobilization on MOFs for the removal of dyes.

### 5.4. The Remediation of Phenols by Enzymes Immobilized on MOFs

Recently, MOFs have gained significant attention as potential solid support for immobilizing enzymes, particularly in the context of eliminating phenolic compounds from industrial wastewater [284]. Among these compounds, BPA, a widely used monomer in polycarbonate plastic production and a known endocrine-disrupting chemical (EDC), has been found in wastewater treatment facilities’ effluents and sludge, highlighting the urgency for effective removal strategies [285].

Catalytic oxidation using laccase, in both homogeneous and heterogeneous forms, is effective in breaking down BPA in wastewater [286]. The introduction of laccase mediators into the system can considerably improve BPA degradation efficiency. For instance, Laccase@HKUST-1 completely decomposed BPA when ABTS was used as the mediator in under 4 h. According to a study by Zhang et al. [234], the transformation of BPA through immobilized laccase was significantly higher than that of free laccase, with immobilized laccase accounting for 74.2% of the transformation compared to 35.5% by free laccase, without taking adsorption into account. The immobilized laccase also displayed a high level of recyclability at 40 °C, with the 75.9% degradation efficiency of BPA remaining after ten cycles.

Laccase encapsulated in Cu-PABA has also been shown to be effective for BPA removal in the laccase/ABTS system [95]. Cu-PABA@Lac demonstrated a limited capacity for BPA removal, achieving only around 26% removal after 12 h in the absence of ABTS, with almost 10% of the removal efficiency attributed to support adsorption. However, the inclusion of ABTS in the system enhanced the degradation of BPA, resulting in the removal of approximately 84.7% of BPA within 12 h. This increase in removal efficiency was largely attributed to the improvement of electron transfer within the system [95].

While incorporating mediators into laccase-based reactions can substantially improve their efficiency, the economic ramifications of utilizing expensive laccase mediators like ABTS must be considered for practical applications [287]. In an effort to create a mediator-free laccase system, researchers combined substrate aptamers with laccase-MOFs hybrids, forming an intelligent biocatalyst known as enzyme-nanoMOFs@aptamers [288]. The incorporation of P1 substrate aptamers into laccase-MOFs composites resulted in the creation of laccase-Cu_3_(BTC)_2_ @P1 nanoflowers (laccase-CBP1), which improved BPA capture, leading to a higher local concentration of BPA around the nanoflowers. This elevated concentration facilitated improved access to the laccase active site, thereby increasing the immobilized laccase’s catalytic activity. Furthermore, CBP1 exhibited laccase-like properties, which, when combined with substrate enrichment, resulted in a 4.4-fold increase in BPA catalytic efficiency, achieving an impressive degradation efficiency of 99.6% for BPA. Furthermore, the reusability, pH tolerance, and thermal stability of the laccase-Cu_3_(BTC)_2_ were improved through surface modification with aptamers, resulting in enhanced BPA removal. The laccase-CBP1 biocatalyst could be conveniently retrieved using magnetically controllable cDNA, and aptamer-BPA binding automatically initiated cyclic enzyme catalysis [288]. MOFs-based laccases have shown effectiveness in degrading other toxic phenolic compounds, such as alkylphenols and 2,4-DCP, without the need for mediators; however, it should be acknowledged that the MOFs support primarily removed these pollutants through adsorption, with laccase-mediated degradation serving a secondary role [238,239].

Enzymatic membrane reactors (EMRs) have been identified as a viable solution for environmental bioremediation. EMRs can be used in batch or continuous mode by combining biocatalytic conversion with membrane separation [289]. Notably, the use of laccase-grafted membranes provides an eco-friendly and economical method for removing persistent organic pollutants, such as phenolic compounds, from wastewater [290]. Researchers have developed highly permeable and effective biocatalytic membranes with immobilized laccase through a three-dimensional modification approach [232]. The integration of water-stable MOFs into the membrane matrix is an effective strategy for increasing laccase loading and enhancing BPA elimination efficacy by providing dual adsorption on both laccase and BPA. The adsorption of the substrate via the biocatalytic membrane limits the enzyme’s entry into the pores, leading to a decrease in pore blockage. As a result, the membrane can degrade 92% of BPA in a single flow-through cycle, requiring a smaller amount of laccase and displaying enhanced reusability [232]. 

Recently, Li et al. [237] described a flexible biocatalytic membrane made of bacterial cellulose (BC), carboxylated-MWCNTs, filter paper, and Lac@ZIF-90. The catalytic membrane displayed outstanding capability in degrading catechol, attributed to the two-fold role of ZIF-90 in preserving the activity of laccase and retaining catechol via adsorption [237]. An EMR assembled using this biocatalytic membrane maintained a high degradation efficiency of about 72% after operating for five days. Similar to this, another EMR that used laccase that was immobilized on a graphene aerogel-Zr-MOFs membrane showed that laccase catalysis and support adsorption together were effective in removing hydroquinone [289]. 

Although EMRs hold significant potential for the treatment of wastewater on large-scale applications, the membrane frequently has short pollutant retention durations as a result of the pressure-driven filtration process [257]. The effectiveness of the biotransformation can be increased by combining MOFs with laccase-based EMRs by extending the time that the substrate is in contact with the laccase. However, MOFs properties like particle size and adsorption capacity strongly influence membrane function [232]. Therefore, careful consideration of MOFs materials is crucial to achieving optimal contaminant removal efficiency for EMRs.

A biocatalytic membrane consisting of Lac@ZIF-90, carboxylated-MWCNTs, bacterial cellulose (BC), and filter paper was recently presented by Li et al. [237]. This membrane demonstrated exceptional catechol degradation performance, owing to ZIF-90’s dual functions of preserving laccase activity and adsorbing catechol [237]. An EMR assembled using this biocatalytic membrane maintained a high degradation efficiency of around 72% after five days of operation. Similarly, an EMR using a graphene aerogel-Zr-MOFs membrane immobilized with laccase was able to effectively eliminate hydroquinone through the combination of laccase and support adsorption. Although EMRs possess considerable promise for extensive wastewater treatment applications, the process of pressure-driven filtration is known to often result in short retention times of pollutants on the membrane surface [289]. The incorporation of laccase-based EMRs with MOFs can extend the interaction duration between laccase and substrate, improving biotransformation efficacy. However, membrane performance largely depends on MOFs characteristics, such as particle size and adsorption capability [232]. Therefore, it is essential to carefully evaluate MOFs materials to obtain the best pollutant removal efficiency for EMRs. To gain more insights into the application of enzyme immobilization on MOFs for removing phenolic compounds, Table 2 can be consulted.

## 6. Challenges and Limitations of Wastewater Treatment Using Immobilized Enzymes 

### 6.1. Costs of Enzyme Immobilization

When assessing enzyme immobilization for wastewater treatment applications, such as eliminating dyes and phenolic compounds, it is crucial to weigh the costs against the potential advantages [291,292,293]. One significant expense is the cost of the support material and the immobilization process itself [294]. Although scientific research usually recommends that the immobilization technique and support should be inexpensive, this notion frequently overlooks critical factors like the support’s loading capacity. Support with greater loading capacity may be more cost-effective, even if it comes at a higher price [295,296,297].

Biocatalysts with low enzyme loading can lead to further issues, such as necessitating a large support mass per reactor volume [298]. When selecting a suitable immobilization system for wastewater treatment, it is essential to consider economic factors, including the cost of the support material, enzyme loading capacity, and operational stability. In cases where enzyme immobilization is required to minimize expenses or enable enzyme reuse, the added value of the product must be balanced against the enzyme cost. Immobilization is only warranted if the immobilized enzyme can achieve the desired reaction yields and reactor productivity [298,299]. The decreasing expense of sustainable products generated through biocatalysis highlights the importance of enzyme recycling and reuse for maintaining low enzyme costs and optimizing cost-effectiveness [293,300,301]. Enzyme immobilization continues to be relevant in wastewater treatment applications, and selecting appropriate immobilization systems is crucial for achieving both cost-effectiveness and efficiency [302,303]. 

### 6.2. The Production of Obstacles That Hinder the Access of Substrates to the Active Site of the Enzyme

This point addresses two main challenges associated with immobilizing enzymes on porous supports for wastewater treatment specifically targeting dyes and phenolic compounds [304]. The first challenge arises when nanoparticles aggregate, creating macro support which has a porous structure, which causes steric obstacles for the enzyme molecules acting on large substrates [305]. The second issue occurs when the enzyme’s active center is not properly oriented with the support surface in mind, leading to steric or partition difficulties for the substrate to access the enzyme activity center [306]. These challenges become more pronounced when dealing with large substrates, such as polysaccharides, proteins, and nucleic acids. In these cases, an enzyme with a correctly oriented active center may be inactive on large substrates while remaining active on smaller ones [306]. Evaluating the overall activity of the biocatalyst can be accomplished by analyzing enzyme activity using both large and small substrates.

It is important to consider the relationship between enzyme activity and enzyme loading when working with large substrates. As support loading increases, enzyme activity against both large and small substrates decreases due to diffusion limitations. At maximum loading, the proximity of immobilized enzyme molecules may prevent the substrate from reaching the active center, resulting in a sudden drop in observed enzyme activity [306]. Extremely high immobilization rates can also contribute to these issues [307,308]. Steric problems can potentially be addressed by employing protocols that allow for different enzyme orientations. Moreover, recent studies indicate that the spatial distribution of enzymes on the support surface can significantly impact the enzyme’s kinetic properties [107].

### 6.3. Using Unstable Supports

Utilizing mechanically unstable supports in wastewater treatment can result in various challenges at both laboratory and industrial scales. Unstable supports may cause biocatalyst particle size to diminish during operation, potentially leading to filter blockages and necessitating the manual removal of material from the reactor. Furthermore, a reduction in particle size can create diffusion and mass transfer issues, impacting enzyme activity or stability [309].

Support fragmentation can also considerably affect biocatalyst performance. For instance, when using co-immobilized enzymes with unstable supports, the enzyme activity ratio may be altered, resulting in decreased final yield and increased unwanted byproducts [310,311]. Researchers should choose supports that are physically compatible with the reactor employed. Another significant problem occurs when support dissolves in the reaction medium, such as improperly cross-linked polymers, complicating downstream product recovery processes. Additionally, the released polymer-carrying enzymes will be flushed out and incorporated into the product, making enzyme reuse unfeasible and affecting filtration systems. To avoid such problems, avoid using supports that may disintegrate in the media and look for alternative supports [312].

### 6.4. The Process of Extrapolating from One Support to Another Is Not Always Straightforward

Adapting immobilization methods from one support material to another can be difficult due to differences in support properties [297]. Several factors should be considered:

- The geometric compatibility between the enzyme and the support can affect enzyme-support interactions. For instance, Eupergit and Sepabead epoxide supports produce different results in enzyme immobilization. [297,309].
- Different supports may allow varying degrees of activation, influencing the enzyme immobilization rate and multi-point covalent linkage. Comparing supports with diverse surface densities of reactive groups may not be fair. The highest activation level should be considered for each support [297].
- Support surfaces can possess unique physical properties, leading to unwanted enzyme–support interactions [313]. Proper blocking can minimize these effects, but physically active supports can never be completely inert. These interactions can impact enzyme function, stability, and the inactivation process [313,314].

To create a novel enzyme immobilization technique, it is advised to begin with an unreactive and water-loving substrate such as agarose and then attempt to replicate the procedure with the desired substrate. Variations can be attributed to the qualities of the substrate, enabling scientists to identify and adjust unsuitable properties of the substrate. In some cases, the active group used for immobilization, rather than the support, generates physical properties that interact with the enzyme [313].

### 6.5. Difficulties in Co-Immobilizing Multiple Enzymes

Developing a co-immobilized enzymatic biocatalyst necessitates the consideration of the arrangement of enzymes within the biocatalyst particle [315]. Achieving the desired enzyme distribution can be difficult, as the simultaneous immobilization of enzymes on the support does not ensure co-localization [315,316]. Some enzymes may immobilize more quickly than others, resulting in varying distributions inside the particle.

To accomplish proper co-localization, researchers should first immobilize the slowest enzyme to ensure its distribution throughout the pores, followed by the faster immobilizing enzyme, which can fill the spaces between the other enzymes’ molecules. This process becomes more complex when using impure enzymes or when the contaminant fractions vary in each batch. Creating concentric enzyme layers is also challenging and can only be guaranteed if certain enzymes immobilize faster than they diffuse [317].

Creating concentric enzyme layers is also challenging. Researchers can only guarantee the formation of a layer of certain enzymes that immobilize faster than they diffuse. When altering the immobilization method or support, studies should be reconducted due to potential changes in enzyme interactions or variations in pore diameter that may impact relative immobilization rates [318]. Particle size changes also necessitate biocatalyst re-optimization due to modified mass transfer. Confocal microscopy with fluorophore-labeled enzymes can be used to examine enzyme localization within the particle [319,320,321]. Despite the complexity of optimization, controlling enzyme order is achievable if researchers comprehend the phenomena taking place in co-immobilization [317].

### 6.6. The Protocols of Immobilization Are Not Complete When All the Enzyme Activity Is Incorporated into the Support

The enzyme immobilization process on support is often mistakenly considered complete by many researchers. However, physically active supports can continue to develop enzyme–support interactions during storage, altering enzyme properties [297]. Although storage conditions can help alleviate this, it is crucial to use inert support for enzyme immobilization [297].

Researchers need to differentiate between immobilization and multi-point covalent attachment. The optimal conditions for immobilization, which dictate enzyme orientation on the support, might not align with the requirements for strong enzyme–support multi-point interactions associated with enzyme and support reactivity [318]. After the initial enzyme immobilization process, it is essential to optimize the subsequent step involving multi-point covalent attachment independently, which may necessitate moderately long reaction times. This approach is critical for achieving full enzyme stabilization through multi-point covalent immobilization using a specific protocol [322]. Emphasizing this point is vital in enzyme immobilization research.

### 6.7. The Utilization of Weakened-Loading Supports

Utilizing immobilized enzymes enables high enzyme concentrations in reactors without the danger of collection, which is crucial for process intensification strategies [323]. However, these benefits can only be achieved with supports that allow high enzyme loadings or loadings appropriate for reaction times and productivity targets [323,324]. Loading capacity is defined by a specific area if the pore size is large enough to allow for enzyme immobilization. Supports with a low specific area (e.g., 1 m^2^ mL^−1^) result in low loadings. Supports with low-stability reactive groups may yield confusing outcomes, as the immobilization rate and final enzyme loading on the support can be influenced by the number of remaining reactive groups [325,326].

Problems may stem from the protein sample components themselves. Employing unpurified enzyme extracts or partially purified commercial enzymes can lead to immobilization issues. For instance, if an enzyme preparation contains large-molecular-weight contaminant proteins, immobilization might be obstructed, as these contaminants can block pores or aggregate, resulting in lower enzyme loadings [309].

The reproducibility of support loading may be impacted by such problems. Solutions include purifying the target enzyme or dissociating multimeric protein complexes before immobilization. In some cases, adding compounds to the immobilization buffer that break down “false” oligomers without affecting the enzyme’s active structure can be beneficial. For example, the use of 1 M urea as an immobilization support was effective in preventing uncontrolled enzyme aggregation of multimeric uridine and purine nucleoside phosphorylases from Bacillus subtilis [327,328].

### 6.8. Enzyme Release from the Support

Enzyme immobilization is essential in diverse applications, such as wastewater treatment for dyes and phenolic compounds. The primary objective of immobilization is to facilitate enzyme recovery and reuse. A significant challenge is preventing enzyme release during operation, as it can affect operational stability and product contamination [323,329,330]. Physical immobilization methods, like ion exchange and hydrophobic supports, can sometimes result in enzyme release due to changes in pH, ionic strength, or reaction products. To minimize enzyme release, researchers can employ support coatings, more hydrophobic supports, or cross-link immobilized enzymes using polymers or covalent bonds [55,331].

Enzyme release is a critical concern when immobilization is assumed to be irreversible but is not, such as when dealing with multimeric enzymes, hetero-functional supports, or supports with strong but reversible enzyme–support bonds. Researchers must carefully evaluate the immobilization method and optimize protocols for each enzyme to minimize enzyme release and ensure optimal immobilization [331].

In conclusion, enzyme immobilization plays a crucial role in various applications, including wastewater treatment. Preventing enzyme release is vital for maintaining operational stability and preventing product contamination. Researchers must consider the immobilization method, the support used, and potential enzyme release mechanisms to design optimal immobilization protocols for each enzyme [329,330,331].

## 7. Conclusions

Enzyme immobilization on emerging and highly porous materials such as graphene, CNTs, and MOFs has proven to be an innovative and promising solution for addressing the removal of dyes and phenolic contaminants from wastewater. This approach effectively overcomes several limitations of traditional methods while providing numerous advantages, such as improved enzyme stability, reusability, and an expanded operational lifetime. The unique physicochemical properties, large specific surface area, and porosity of graphene, CNTs, and MOFs facilitate optimal enzyme–substrate interactions, leading to the enhanced removal of organic water contaminants. Moreover, these materials offer the possibility of tailoring their properties, allowing for the creation of customized solutions to address specific wastewater treatment challenges, ultimately allowing for more targeted and effective remediation strategies.

Despite the high potential and promises of enzyme immobilization on graphene, CNTs, and MOFs in boosting the efficacy of enzymatic wastewater treatment, several challenges remain unresolved, including scaling up the production of biocatalytic nanoparticles, maintaining quality consistency, optimizing enzyme immobilization and operational parameters for maximum efficiency, and evaluating the long-term stability and environmental impact of the enzyme immobilization process and the utilized supports. Nonetheless, the adoption of enzyme immobilization on emerging materials such as graphene, CNTs, and MOFs represents a valuable and promising approach in wastewater treatment. To ensure the successful large-scale applications of this emerging technology, it is vital to continuously explore and address the associated challenges. Advancements in this area hold the potential to substantially influence the wastewater treatment industry, fostering the development of more efficient, sustainable, and environmentally friendly solutions to combat the growing water pollution problem.

To guide the future direction of research in this field, a few key areas merit particular attention:

1. Hybrid Materials: Given the unique properties of graphene, CNTs, and MOFs, the development of hybrid materials that combine their strengths could lead to superior supports for enzyme immobilization [221,222]. Future research could explore this avenue and potentially unveil highly efficient, tailor-made materials for wastewater treatment.
2. Enzyme-Substrate Dynamics: While we have discussed the enzyme-substrate interactions in the context of the physicochemical properties of graphene, CNTs, and MOFs, further understanding of these dynamics in various operational conditions will enhance the efficacy of the treatment process [83,100,102]. Unraveling these complex interactions could provide critical insights into the design of advanced immobilization techniques.
3. Environmental Impacts: Long-term environmental studies are needed to ensure the sustainability of using these emerging materials in the enzyme immobilization process. The environmental fate of these materials, once they complete their operational lifecycle, is still not well understood and requires thorough investigation.
4. Cost-Effectiveness: The economic aspect of implementing these emerging materials in real-world scenarios is another research gap that needs to be addressed. It will be important to develop techniques to lower the cost of producing and using these materials, ensuring their feasibility for industrial applications.

Addressing these areas in future research will provide comprehensive insights into the practical applicability and sustainability of enzyme immobilization on emerging materials such as graphene, CNTs, and MOFs for wastewater treatment. This, in turn, will foster the development of more efficient, sustainable, and environmentally friendly solutions to combat the water pollution problem.

## Data Availability

Data are contained within the article. For any additional data request, the authors may be contacted.

## References

[1] Farhan Hanafi M., Sapawe N. (2020). A Review on the Water Problem Associate with Organic Pollutants Derived from Phenol, Methyl Orange, and Remazol Brilliant Blue Dyes. Mater. Today Proc..

[2] Lellis B., Fávaro-Polonio C.Z., Pamphile J.A., Polonio J.C. (2019). Effects of Textile Dyes on Health and the Environment and Bioremediation Potential of Living Organisms. Biotechnol. Res. Innov..

[3] Asmaly H.A., Abussaud B., Ihsanullah, Saleh T.A., Bukhari A.A., Laoui T., Shemsi A.M., Gupta V.K., Atieh M.A. (2015). Evaluation of Micro- and Nano-Carbon-Based Adsorbents for the Removal of Phenol from Aqueous Solutions. Toxicol. Environ. Chem..

[4] Asmaly H.A., Ihsanullah, Abussaud B., Saleh T.A., Laoui T., Gupta V.K., Atieh M.A. (2016). Adsorption of Phenol on Aluminum Oxide Impregnated Fly Ash. Desalin. Water Treat..

[5] Ahmed J., Thakur A., Goyal A. (2021). Industrial Wastewater and Its Toxic Effects. Biological Treatment of Industrial Wastewater.

[6] Muthukumaran M. (2021). Advances in Bioremediation of Nonaqueous Phase Liquid Pollution in Soil and Water. Biological Approaches to Controlling Pollutants.

[7] Landrigan P.J., Stegeman J.J., Fleming L.E., Allemand D., Anderson D.M., Backer L.C., Brucker-Davis F., Chevalier N., Corra L., Czerucka D. (2020). Human Health and Ocean Pollution. Ann. Glob. Health.

[8] Anku W.W., Mamo M.A., Govender P.P. (2017). Phenolic Compounds in Water: Sources, Reactivity, Toxicity and Treatment Methods. Phenolic Compounds—Natural Sources, Importance and Applications.

[9] Manasa R.L., Mehta A. (2020). Wastewater: Sources of Pollutants and Its Remediation. Environ. Chem. A Sustain. World.

[10] Akpor O.B., Ohiobor G.O., Olaolu T.D., Oghenerobor B., Akpor G.O., Ohiobor T., Debby O. (2014). Heavy Metal Pollutants in Wastewater Effluents: Sources, Effects and Remediation. Adv. Biosci. Bioeng..

[11] Ahmed A., Forster M., Jin J., Myers P., Zhang H. (2015). Tuning Morphology of Nanostructured ZIF-8 on Silica Microspheres and Applications in Liquid Chromatography and Dye Degradation. ACS Appl. Mater. Interfaces.

[12] Gao W., Fatehi P. (2018). Fly Ash Based Adsorbent for Treating Bleaching Effluent of Kraft Pulping Process. Sep. Purif. Technol..

[13] Li G., Xu Q., Jin X., Li R., Dharmarajan R., Chen Z. (2018). Enhanced Adsorption and Fenton Oxidation of 2,4-Dichlorophenol in Aqueous Solution Using Organobentonite Supported NZVI. Sep. Purif. Technol..

[14] Abussaud B., Asmaly H.A., Ihsanullah, Saleh T.A., Gupta V.K., Laoui T., Atieh M.A. (2016). Sorption of Phenol from Waters on Activated Carbon Impregnated with Iron Oxide, Aluminum Oxide and Titanium Oxide. J. Mol. Liq..

[15] Bahadi S.A., Iddrisu M., Al-Sakkaf M.K., Elgzoly M.A.A., Drmosh Q.A., Al-Amrani W.A., Ahmed U., Zahid U., Onaizi S.A. (2023). Optimization of Methyl Orange Adsorption on MgFeAl-LTH through the Manipulation of Solution Chemistry and Synthesis Conditions. Emerg. Mater..

[16] Bahadi S.A., Iddrisu M., Al-Sakkaf M.K., Elgzoly M.A.A., Al-Amrani W.A., Ahmed U., Zahid U., Drmosh Q.A., Onaizi S.A. (2023). Chemically versus Thermally Reduced Graphene Oxide: Effects of Reduction Methods and Reducing Agents on the Adsorption of Phenolic Compounds from Wastewater. Emerg. Mater..

[17] Ismail U.M., Onaizi S.A., Vohra M.S. (2023). Novel MgCuAl-Layered Triple Hydroxide for Aqueous Selenite and Selenate Treatment. Emerg. Mater..

[18] Fortunato L., Elcik H., Blankert B., Ghaffour N., Vrouwenvelder J. (2021). Textile Dye Wastewater Treatment by Direct Contact Membrane Distillation: Membrane Performance and Detailed Fouling Analysis. J. Memb. Sci..

[19] Criscuoli A., Zhong J., Figoli A., Carnevale M.C., Huang R., Drioli E. (2008). Treatment of Dye Solutions by Vacuum Membrane Distillation. Water Res..

[20] Jaradat A.Q., Gharaibeh S., Abu Irjei M. (2018). The Application of Solar Distillation Technique as a Mean for Olive Mill Wastewater Management. Water Environ. J..

[21] Crini G., Lichtfouse E. (2018). Wastewater Treatment: An Overview. Green Adsorbents for Pollutant Removal.

[22] Shikuku V.O., Nyairo W.N. (2022). Advanced Oxidation Processes for Dye Removal from Wastewater. Impact of Textile Dyes on Public Health and the Environment.

[23] Loos G., Scheers T., Van Eyck K., Van Schepdael A., Adams E., Van der Bruggen B., Cabooter D., Dewil R. (2018). Electrochemical Oxidation of Key Pharmaceuticals Using a Boron Doped Diamond Electrode. Sep. Purif. Technol..

[24] Liu Z., Meng H., Zhang H., Cao J., Zhou K., Lian J. (2018). Highly Efficient Degradation of Phenol Wastewater by Microwave Induced H_2_O_2_-CuOx/GAC Catalytic Oxidation Process. Sep. Purif. Technol..

[25] Pandit P., Basu S. (2004). Dye and Solvent Recovery in Solvent Extraction Using Reverse Micelles for the Removal of Ionic Dyes. Ind. Eng. Chem. Res..

[26] Pandit P., Basu S. (2004). Removal of Ionic Dyes from Water by Solvent Extraction Using Reverse Micelles. Environ. Sci. Technol..

[27] Asrami M.R., Saien J. (2018). Salting-out Effect on Extraction of Phenol from Aqueous Solutions by [Hmim][NTf2] Ionic Liquid: Experimental Investigations and Modeling. Sep. Purif. Technol..

[28] González E.J., Díaz I., Gonzalez-Miquel M., Rodríguez M., Sueiras A. (2018). On the Behavior of Imidazolium versus Pyrrolidinium Ionic Liquids as Extractants of Phenolic Compounds from Water: Experimental and Computational Analysis. Sep. Purif. Technol..

[29] Kadhim R.J., Al-Ani F.H., Al-Shaeli M., Alsalhy Q.F., Figoli A. (2020). Removal of Dyes Using Graphene Oxide (GO) Mixed Matrix Membranes. Membranes.

[30] Ouyang Z., Huang Z., Tang X., Xiong C., Tang M., Lu Y. (2019). A Dually Charged Nanofiltration Membrane by PH-Responsive Polydopamine for Pharmaceuticals and Personal Care Products Removal. Sep. Purif. Technol..

[31] Zhang Y., Yu W., Li R., Xu Y., Shen L., Lin H., Liao B.Q., Wu G. (2019). Novel Conductive Membranes Breaking through the Selectivity-Permeability Trade-off for Congo Red Removal. Sep. Purif. Technol..

[32] Sunil K., Sherugar P., Rao S., Lavanya C., Balakrishna G.R., Arthanareeswaran G., Padaki M. (2021). Prolific Approach for the Removal of Dyes by an Effective Interaction with Polymer Matrix Using Ultrafiltration Membrane. J. Environ. Chem. Eng..

[33] Badvi K., Javanbakht V. (2021). Enhanced Photocatalytic Degradation of Dye Contaminants with TiO_2_ Immobilized on ZSM-5 Zeolite Modified with Nickel Nanoparticles. J. Clean. Prod..

[34] Sirirerkratana K., Kemacheevakul P., Chuangchote S. (2019). Color Removal from Wastewater by Photocatalytic Process Using Titanium Dioxide-Coated Glass, Ceramic Tile, and Stainless Steel Sheets. J. Clean. Prod..

[35] Nguyen D.C.T., Cho K.Y., Oh W.C. (2019). Mesoporous CuO-Graphene Coating of Mesoporous TiO_2_ for Enhanced Visible-Light Photocatalytic Activity of Organic Dyes. Sep. Purif. Technol..

[36] Lin J.C.T., Sopajaree K., Jitjanesuwan T., Lu M.C. (2018). Application of Visible Light on Copper-Doped Titanium Dioxide Catalyzing Degradation of Chlorophenols. Sep. Purif. Technol..

[37] Alkadhem A.M., Elgzoly M.A.A., Onaizi S.A. (2020). Novel Amine-Functionalized Magnesium Oxide Adsorbents for CO_2_ Capture at Ambient Conditions. J. Environ. Chem. Eng..

[38] Hezam A., Drmosh Q.A., Ponnamma D., Bajiri M.A., Qamar M., Namratha K., Zare M., Nayan M.B., Onaizi S.A., Byrappa K. (2022). Strategies to Enhance ZnO Photocatalyst’s Performance for Water Treatment: A Comprehensive Review. Chem. Rec..

[39] Al Lagtah N.M.A., Onaizi S.A., Albadarin A.B., Ghaith F.A., Nour M.I. (2019). Techno-Economic Analysis of the Effects of Heat Integration and Different Carbon Capture Technologies on the Performance of Coal-Based IGCC Power Plants. J. Environ. Chem. Eng..

[40] Almarouf H.S., Nasser M.S., Al-Marri M.J., Khraisheh M., Onaizi S.A. (2015). Demulsification of Stable Emulsions from Produced Water Using a Phase Separator with Inclined Parallel Arc Coalescing Plates. J. Pet. Sci. Eng..

[41] Al-Sakkaf M.K., Onaizi S.A. (2023). Crude Oil/Water Nanoemulsions Stabilized by Rhamnolipid Biosurfactant: Effects of Acidity/Basicity and Salinity on Emulsion Characteristics, Stability, and Demulsification. Fuel.

[42] Lateef S.A., Ajumobi O.O., Onaizi S.A. (2019). Enzymatic Desulfurization of Crude Oil and Its Fractions: A Mini Review on the Recent Progresses and Challenges. Arab. J. Sci. Eng..

[43] Al-Sakkaf M.K., Onaizi S.A. (2022). Rheology, Characteristics, Stability, and PH-Responsiveness of Biosurfactant-Stabilized Crude Oil/Water Nanoemulsions. Fuel.

[44] Onaizi S.A., Alsulaimani M., Al-Sakkaf M.K., Bahadi S.A., Mahmoud M., Alshami A. (2021). Crude Oil/Water Nanoemulsions Stabilized by Biosurfactant: Stability and PH-Switchability. J. Pet. Sci. Eng..

[45] Onaizi S.A. (2021). Demulsification of Crude Oil/Water Nanoemulsions Stabilized by Rhamnolipid Biosurfactant Using Enzymes and PH-Swing. Sep. Purif. Technol..

[46] Onaizi S.A., He L., Middelberg A.P.J. (2010). The Construction, Fouling and Enzymatic Cleaning of a Textile Dye Surface. J. Colloid Interface Sci..

[47] Onaizi S.A. (2018). Dynamic Surface Tension and Adsorption Mechanism of Surfactin Biosurfactant at the Air–Water Interface. Eur. Biophys. J..

[48] Onaizi S.A., Malcolm A.S., He L., Middelberg A.P.J. (2007). Directed Disassembly of an Interfacial Rubisco Protein Network. Langmuir.

[49] Singh N., Singh J. (2002). An Enzymatic Method for Removal of Phenol from Industrial Effluent. Prep. Biochem. Biotechnol..

[50] Ariaeenejad S., Motamedi E., Salekdeh G.H. (2022). Highly Efficient Removal of Dyes from Wastewater Using Nanocellulose from Quinoa Husk as a Carrier for Immobilization of Laccase. Bioresour. Technol..

[51] Sariaslani F.S., Dalton H. (1989). Microbial Enzymes for Oxidation of Organic Molecules. Crit. Rev. Biotechnol..

[52] Misal S.A., Gawai K.R. (2018). Azoreductase: A Key Player of Xenobiotic Metabolism. Bioresour. Bioprocess..

[53] Gaur G., Gänzle M.G. (2023). Conversion of (Poly)Phenolic Compounds in Food Fermentations by Lactic Acid Bacteria: Novel Insights into Metabolic Pathways and Functional Metabolites. Curr. Res. Food Sci..

[54] Basso A., Serban S. (2019). Industrial Applications of Immobilized Enzymes—A Review. Mol. Catal..

[55] Jesionowski T., Zdarta J., Krajewska B. (2014). Enzyme Immobilization by Adsorption: A Review. Adsorption.

[56] Alshabib M., Onaizi S.A. (2019). A Review on Phenolic Wastewater Remediation Using Homogeneous and Heterogeneous Enzymatic Processes: Current Status and Potential Challenges. Sep. Purif. Technol..

[57] Torres J.A., Nogueira F.G.E., Silva M.C., Lopes J.H., Tavares T.S., Ramalho T.C., Corrêa A.D. (2017). Novel Eco-Friendly Biocatalyst: Soybean Peroxidase Immobilized onto Activated Carbon Obtained from Agricultural Waste. RSC Adv..

[58] Gholami-Borujeni F., Mahvi A.H., Naseri S., Faramarzi M.A., Nabizadeh R., Alimohammadi M. (2011). Application of Immobilized Horseradish Peroxidase for Removal and Detoxification of Azo Dye from Aqueous Solution. Res. J. Chem. Environ..

[59] Silva M.C., Torres J.A., Vasconcelos De Sá L.R., Chagas P.M.B., Ferreira-Leitão V.S., Corrêa A.D. (2013). The Use of Soybean Peroxidase in the Decolourization of Remazol Brilliant Blue R and Toxicological Evaluation of Its Degradation Products. J. Mol. Catal. B Enzym..

[60] Chiong T., Lau S.Y., Lek Z.H., Koh B.Y., Danquah M.K. (2016). Enzymatic Treatment of Methyl Orange Dye in Synthetic Wastewater by Plant-Based Peroxidase Enzymes. J. Environ. Chem. Eng..

[61] Bhatia D., Sharma N.R., Singh J., Kanwar R.S. (2017). Biological Methods for Textile Dye Removal from Wastewater: A Review. Crit. Rev. Environ. Sci. Technol..

[62] Forootanfar H., Moezzi A., Aghaie-Khozani M., Mahmoudjanlou Y., Ameri A., Niknejad F., Ali Faramarzi M. (2012). Synthetic Dye Decolorization by Three Sources of Fungal Laccase. Iran. J. Environ. Health Sci. Eng..

[63] Onaizi S.A., Alshabib M. (2021). The Degradation of Bisphenol A by Laccase: Effect of Biosurfactant Addition on the Reaction Kinetics under Various Conditions. Sep. Purif. Technol..

[64] Silva M.C., Torres J.A., Castro A.A., da Cunha E.F.F., Alves de Oliveira L.C., Corrêa A.D., Ramalho T.C. (2016). Combined Experimental and Theoretical Study on the Removal of Pollutant Compounds by Peroxidases: Affinity and Reactivity toward a Bioremediation Catalyst. J. Biomol. Struct. Dyn..

[65] Veitch N.C. (2004). Horseradish Peroxidase: A Modern View of a Classic Enzyme. Phytochemistry.

[66] Jaiswal N., Pandey V.P., Dwivedi U.N. (2016). Immobilization of Papaya Laccase in Chitosan Led to Improved Multipronged Stability and Dye Discoloration. Int. J. Biol. Macromol..

[67] Effron D., De La Horra A.M., Defrieri R.L., Fontanive V., Palma R.M. (2006). Effect of Cadmium, Copper, and Lead on Different Enzyme Activities in a Native Forest Soil. Commun. Soil Sci. Plant Anal..

[68] Torres J.A., Silva M.C., Lopes J.H., Nogueira A.E., Nogueira F.G.E., Corrêa A.D. (2018). Development of a Reusable and Sustainable Biocatalyst by Immobilization of Soybean Peroxidase onto Magnetic Adsorbent. Int. J. Biol. Macromol..

[69] Wang C., Zhang H., Ren D., Li Q., Zhang S., Feng T. (2015). Effect of Direct-Current Electric Field on Enzymatic Activity and the Concentration of Laccase. Indian. J. Microbiol..

[70] Alshabib M., Onaizi S.A. (2020). Enzymatic Remediation of Bisphenol A from Wastewaters: Effects of Biosurfactant, Anionic, Cationic, Nonionic, and Polymeric Additives. Water Air Soil. Pollut..

[71] Alshabib M., Onaizi S.A. (2019). Effects of Surface Active Additives on the Enzymatic Treatment of Phenol and Its Derivatives: A Mini Review. Curr. Pollut. Rep..

[72] Sellami K., Couvert A., Nasrallah N., Maachi R., Tandjaoui N., Abouseoud M., Amrane A. (2021). Bio-Based and Cost Effective Method for Phenolic Compounds Removal Using Cross-Linked Enzyme Aggregates. J. Hazard. Mater..

[73] Villegas L.G.C., Mashhadi N., Chen M., Mukherjee D., Taylor K.E., Biswas N. (2016). A Short Review of Techniques for Phenol Removal from Wastewater. Curr. Pollut. Rep..

[74] Onaizi S.A. (2021). Statistical Analyses of the Effect of Rhamnolipid Biosurfactant Addition on the Enzymatic Removal of Bisphenol A from Wastewater. Biocatal. Agric. Biotechnol..

[75] Onaizi S.A. Enzymatic Treatment of Phenolic Wastewater: Effects of Salinity and Biosurfactant Addition. Proceedings of the International Petroleum Technology Conference.

[76] Chatterjee S., Kumari S., Rath S., Das S. (2022). Prospects and Scope of Microbial Bioremediation for the Restoration of the Contaminated Sites. Microbial Biodegradation and Bioremediation.

[77] Kanaujiya D.K., Paul T., Sinharoy A., Pakshirajan K. (2019). Biological Treatment Processes for the Removal of Organic Micropollutants from Wastewater: A Review. Curr. Pollut. Rep..

[78] Ramirez E., de la Luz Asunción M., Rivalcoba V.S., Hernández A., Santos C.V. (2017). Removal of Phenolic Compounds from Water by Adsorption and Photocatalysis. Phenolic Compounds—Natural Sources, Importance and Applications.

[79] Betancur-Ramírez K.J., Meneses-Jácome A., Ruiz-Colorado A.A., Gallego-Suárez D. (2021). Life Cycle Assessment of an Alternative Enzymatic-Biological Treatment for Effluents from Industrial Processing of Potatoes. J. Clean. Prod..

[80] Sirisha V.L., Jain A., Jain A. (2016). Enzyme Immobilization: An Overview on Methods, Support Material, and Applications of Immobilized Enzymes. Adv. Food Nutr. Res..

[81] Lou W.Y., Fernández-Lucas J., Ge J., Wu C. (2021). Enzyme or Whole Cell Immobilization for Efficient Biocatalysis: Focusing on Novel Supporting Platforms and Immobilization Techniques. Front. Bioeng. Biotechnol..

[82] Saravanan A., Kumar P.S., Vo D.V.N., Jeevanantham S., Karishma S., Yaashikaa P.R. (2021). A Review on Catalytic-Enzyme Degradation of Toxic Environmental Pollutants: Microbial Enzymes. J. Hazard. Mater..

[83] Xu K., Chen X., Zheng R., Zheng Y. (2020). Immobilization of Multi-Enzymes on Support Materials for Efficient Biocatalysis. Front. Bioeng. Biotechnol..

[84] Irfan M., Ghazanfar M., Ur Rehman A., Siddique A. (2019). Strategies to Reuse Cellulase: Immobilization of Enzymes (Part II). Approaches to Enhance Industrial Production of Fungal Cellulases.

[85] Sepahvand H., Heravi M.M., Saber M., Hooshmand S.E. (2022). Techniques and Support Materials for Enzyme Immobilization Using Ugi Multicomponent Reaction: An Overview. J. Iran. Chem. Soc..

[86] Wang Y., Gu Y., Yang S. (2022). Developing a Novel Strategy for Light-Triggered Reversible Enzyme Immobilization and Reuse of Support. Alex. Eng. J..

[87] Rodrigues R.C., Ortiz C., Berenguer-Murcia Á., Torres R., Fernández-Lafuente R. (2013). Modifying Enzyme Activity and Selectivity by Immobilization. Chem. Soc. Rev..

[88] Lanie M., Ab H. (2021). Enzymatic Strategies for Asymmetric Synthesis. RSC Chem. Biol..

[89] Nguyen L.N., Hai F.I., Dosseto A., Richardson C., Price W.E., Nghiem L.D. (2016). Continuous Adsorption and Biotransformation of Micropollutants by Granular Activated Carbon-Bound Laccase in a Packed-Bed Enzyme Reactor. Bioresour. Technol..

[90] Isanapong J., Lohawet K., Kumnorkaew P. (2021). Optimization and Characterization of Immobilized Laccase on Titanium Dioxide Nanostructure and Its Application in Removal of Remazol Brilliant Blue R. Biocatal. Agric. Biotechnol..

[91] Ponmudi K., Cherian A.R., Varghese A. (2023). Carbon Dots as an Effective Material in Enzyme Immobilization for Sensing Applications. Carbon Dots in Analytical Chemistry Detection and Imaging.

[92] Oliveira F.L., França A.d.S., de Castro A.M., Alves de Souza R.O.M., Esteves P.M., Gonçalves R.S.B. (2020). Enzyme Immobilization in Covalent Organic Frameworks: Strategies and Applications in Biocatalysis. Chempluschem.

[93] Zhang J., Zhang J., Zhang F., Yang H., Huang X., Liu H., Guo S. (2010). Graphene Oxide as a Matrix for Enzyme Immobilization. Langmuir.

[94] Zhang W., Yang Q., Luo Q., Shi L., Meng S. (2020). Laccase-Carbon Nanotube Nanocomposites for Enhancing Dyes Removal. J. Clean. Prod..

[95] Yuan Y., Cai W., Xu J., Cheng J., Du K.S. (2021). Recyclable Laccase by Coprecipitation with Aciduric Cu-Based MOFs for Bisphenol A Degradation in an Aqueous Environment. Colloids Surf. B Biointerfaces.

[96] Nawaz A.F., Zafar S., Fatim S.L., Shahzadi K., Fatima Z., Siddique I. (2020). Use of Nanomaterials for the Immobilization of Industrially Important Enzymes. J. Nanotechnol. Res..

[97] Soares A.M.B.F., Gonçalves L.M.O., Ferreira R.D.S., de Souza J.M., Fangueiro R., Alves M.M.M., Carvalho F.A.A., Mendes A.N., Cantanhêde W. (2020). Immobilization of Papain Enzyme on a Hybrid Support Containing Zinc Oxide Nanoparticles and Chitosan for Clinical Applications. Carbohydr. Polym..

[98] Ramakrishna T.R.B., Nalder T.D., Yang W., Marshall S.N., Barrow C.J. (2018). Controlling Enzyme Function through Immobilisation on Graphene, Graphene Derivatives and Other Two Dimensional Nanomaterials. J. Mater. Chem. B.

[99] Wen H., Nallathambi V., Chakraborty D., Barton S.C. (2011). Carbon Fiber Microelectrodes Modified with Carbon Nanotubes as a New Support for Immobilization of Glucose Oxidase. Microchim. Acta.

[100] Thakur K., Attri C., Seth A. (2021). Nanocarriers-Based Immobilization of Enzymes for Industrial Application. 3 Biotech..

[101] Li S.F., Zhai X.J., Zhang C., Mo H.L., Zang S.Q. (2020). Enzyme Immobilization in Highly Ordered Macro–Microporous Metal–Organic Frameworks for Rapid Biodegradation of Hazardous Dyes. Inorg. Chem. Front..

[102] Feng L., Wang K.Y., Lv X.L., Yan T.H., Zhou H.C. (2020). Hierarchically Porous Metal–Organic Frameworks: Synthetic Strategies and Applications. Natl. Sci. Rev..

[103] Guisan J.M., López-Gallego F., Bolivar J.M., Rocha-Martín J., Fernandez-Lorente G. (2020). The Science of Enzyme Immobilization. Methods Mol. Biol..

[104] Khan M.R. (2021). Immobilized Enzymes: A Comprehensive Review. Bull. Natl. Res. Cent..

[105] Imam H.T., Marr P.C., Marr A.C. (2021). Enzyme Entrapment, Biocatalyst Immobilization without Covalent Attachment. Green. Chem..

[106] Brena B., González-Pombo P., Batista-Viera F. (2013). Immobilization of Enzymes and Cells.

[107] Spahn C., Minteer S.D. (2008). Enzyme Immobilization in Biotechnology. Recent. Pat. Eng..

[108] Dey G., Nagpal V., Banerjee R. (2002). Immobilization of Alpha-Amylase from Bacillus Circulans GRS 313 on Coconut Fiber. Appl. Biochem. Biotechnol..

[109] Rosales-Hernández M., Kispert L., Torres-Ramírez E., Ramírez-Rosales D., Zamorano-Ulloa R., Trujillo-Ferrara J. (2007). Electron Paramagnetic Resonance Analyses of Biotransformation Reactions with Cytochrome P-450 Immobilized on Mesoporous Molecular Sieves. Biotechnol. Lett..

[110] Karagulyan H.K., Gasparyan V.K., Decker S.R. (2008). Immobilization of Fungal Beta-Glucosidase on Silica Gel and Kaolin Carriers. Appl. Biochem. Biotechnol..

[111] Brígida A.I.S., Calado V.M.A., Gonçalves L.R.B., Coelho M.A.Z. (2010). Effect of Chemical Treatments on Properties of Green Coconut Fiber. Carbohydr. Polym..

[112] Huang X.J., Chen P.C., Huang F., Ou Y., Chen M.R., Xu Z.K. (2011). Immobilization of Candida Rugosa Lipase on Electrospun Cellulose Nanofiber Membrane. J. Mol. Catal. B Enzym..

[113] Mitchell S., Pérez-Ramírez J. (2011). Mesoporous Zeolites as Enzyme Carriers: Synthesis, Characterization, and Application in Biocatalysis. Catal. Today.

[114] Díaz J.F., Balkus K.J. (1996). Enzyme Immobilization in MCM-41 Molecular Sieve. J. Mol. Catal. B Enzym..

[115] Persson M., Wehtje E., Adlercreutz P. (2000). Immobilisation of Lipases by Adsorption and Deposition: High Protein Loading Gives Lower Water Activity Optimum. Biotechnol. Lett..

[116] Sabbani S., Hedenström E., Nordin O. (2006). The Enantioselectivity of Candida Rugosa Lipase Is Influenced by the Particle Size of the Immobilising Support Material Accurel. J. Mol. Catal. B Enzym..

[117] Cunha A.G., Fernández-Lorente G., Bevilaqua J.V., Destain J., Paiva L.M.C., Freire D.M.G., Fernández-Lafuente R., Guisán J.M. (2008). Immobilization of Yarrowia Lipolytica Lipase—A Comparison of Stability of Physical Adsorption and Covalent Attachment Techniques. Appl. Biochem. Biotechnol..

[118] Cabrera-Padilla R.Y., Lisboa M.C., Fricks A.T., Franceschi E., Lima A.S., Silva D.P., Soares C.M.F. (2012). Immobilization of Candida Rugosa Lipase on Poly(3-hydroxybutyrate-co-hydroxyvalerate): A New Eco-Friendly Support. J. Ind. Microbiol. Biotechnol..

[119] Mishra N., Pithawala K., Bahadur A. (2011). Byssus Thread: A Novel Support Material for Urease Immobilization. Appl. Biochem. Biotechnol..

[120] Popat A., Hartono S.B., Stahr F., Liu J., Qiao S.Z., Lu G.Q. (2011). Mesoporous Silica Nanoparticles for Bioadsorption, Enzyme Immobilisation, and Delivery Carriers. Nanoscale.

[121] Magner E. (2013). Immobilisation of Enzymes on Mesoporous Silicate Materials. Chem. Soc. Rev..

[122] Zucca P., Sanjust E. (2014). Inorganic Materials as Supports for Covalent Enzyme Immobilization: Methods and Mechanisms. Molecules.

[123] Azodi M., Falamaki C., Mohsenifar A. (2011). Sucrose Hydrolysis by Invertase Immobilized on Functionalized Porous Silicon. J. Mol. Catal. B Enzym..

[124] Homaei A.A., Sariri R., Vianello F., Stevanato R. (2013). Enzyme Immobilization: An Update. J. Chem. Biol..

[125] Hanefeld U., Gardossi L., Magner E. (2009). Understanding Enzyme Immobilisation. Chem. Soc. Rev..

[126] Betancor L., Luckarift H.R. (2008). Bioinspired Enzyme Encapsulation for Biocatalysis. Trends Biotechnol..

[127] Kurzbaum E., Raizner Y., Kuc M.E., Kulikov A., Hakimi B., Kruh L.I., Menashe O. (2019). Phenol Biodegradation by Bacterial Cultures Encapsulated in 3D Microfiltration-Membrane Capsules. Environ. Technol..

[128] Maghraby Y.R., El-Shabasy R.M., Ibrahim A.H., Azzazy H.M.E.S. (2023). Enzyme Immobilization Technologies and Industrial Applications. ACS Omega.

[129] Song J., He W., Shen H., Zhou Z., Li M., Su P., Yang Y. (2019). Construction of Multiple Enzyme Metal–Organic Frameworks Biocatalyst via DNA Scaffold: A Promising Strategy for Enzyme Encapsulation. Chem. Eng. J..

[130] Tran D.N., Balkus K.J. (2011). Perspective of Recent Progress in Immobilization of Enzymes. ACS Catal..

[131] Datta S., Christena L.R., Rajaram Y.R.S. (2013). Enzyme Immobilization: An Overview on Techniques and Support Materials. 3 Biotech..

[132] Hartmann M., Jung D. (2010). Biocatalysis with Enzymes Immobilized on Mesoporous Hosts: The Status Quo and Future Trends. J. Mater. Chem..

[133] Lôpez-Serrano P., Cao L., Van Rantwijk F., Sheldon R.A. (2002). Cross-Linked Enzyme Aggregates with Enhanced Activity: Application to Lipases. Biotechnol. Lett..

[134] Aytar B.S., Bakir U. (2008). Preparation of Cross-Linked Tyrosinase Aggregates. Process Biochem..

[135] Roessl U., Nahálka J., Nidetzky B. (2010). Carrier-Free Immobilized Enzymes for Biocatalysis. Biotechnol. Lett..

[136] Singh B.D. (2012). Biotechnology Expanding Horizons.

[137] Shen Q., Yang R., Hua X., Ye F., Zhang W., Zhao W. (2011). Gelatin-Templated Biomimetic Calcification for β-Galactosidase Immobilization. Process Biochem..

[138] Ispas C., Sokolov I., Andreescu S. (2009). Enzyme-Functionalized Mesoporous Silica for Bioanalytical Applications. Anal. Bioanal. Chem..

[139] Chen G.C., Kuan I.C., Hong J.R., Tsai B.H., Lee S.L., Yu C.Y. (2011). Activity Enhancement and Stabilization of Lipase from Pseudomonas Cepacia in Polyallylamine-Mediated Biomimetic Silica. Biotechnol. Lett..

[140] Tümtürk H., Karaca N., Demirel G., Şahin F. (2007). Preparation and Application of Poly(N,N-Dimethylacrylamide-Co-Acrylamide) and Poly(N-Isopropylacrylamide-Co-Acrylamide)/Kappa-Carrageenan Hydrogels for Immobilization of Lipase. Int. J. Biol. Macromol..

[141] Jegannathan K.R., Jun-Yee L., Chan E.S., Ravindra P. (2010). Production of Biodiesel from Palm Oil Using Liquid Core Lipase Encapsulated in κ-Carrageenan. Fuel.

[142] Brena B., González-Pombo P., Batista-Viera F. (2013). Immobilization of Enzymes: A Literature Survey. Methods Mol. Biol..

[143] Patil J.S., Kamalapur M.V., Marapur S.C., Kadam D.V. (2010). Ionotropic gelation and polyelectrolyte complexation: The novel techniques to design hydrogel particulate sustained, modulated drug delivery system: A review. Dig. J. Nanomater. Biostruct..

[144] Rother C., Nidetzky B. (2014). Enzyme Immobilization by Microencapsulation: Methods, Materials, and Technological Applications. Encyclopedia of Industrial Biotechnology.

[145] Honda T., Miyazaki M., Nakamura H., Maeda H. (2005). Immobilization of Enzymes on a Microchannel Surface through Cross-Linking Polymerization. Chem. Commun..

[146] Fu J., Reinhold J., Woodbury N.W. (2011). Peptide-Modified Surfaces for Enzyme Immobilization. PLoS ONE.

[147] Hsieh H.J., Liu P.C., Liao W.J. (2000). Immobilization of Invertase via Carbohydrate Moiety on Chitosan to Enhance Its Thermal Stability. Biotechnol. Lett..

[148] Szymańska K., Bryjak J., Jarzębski A.B. (2009). Immobilization of Invertase on Mesoporous Silicas to Obtain Hyper Active Biocatalysts. Top. Catal..

[149] Kim J., Jia H., Wang P. (2006). Challenges in Biocatalysis for Enzyme-Based Biofuel Cells. Biotechnol. Adv..

[150] Huang X.J., Yu A.G., Xu Z.K. (2008). Covalent Immobilization of Lipase from Candida Rugosa onto Poly(acrylonitrile-co-2-hydroxyethyl methacrylate) Electrospun Fibrous Membranes for Potential Bioreactor Application. Bioresour. Technol..

[151] Sakai S., Liu Y., Yamaguchi T., Watanabe R., Kawabe M., Kawakami K. (2010). Immobilization of Pseudomonas Cepacia Lipase onto Electrospun Polyacrylonitrile Fibers through Physical Adsorption and Application to Transesterification in Nonaqueous Solvent. Biotechnol. Lett..

[152] Wu L., Yuan X., Sheng J. (2005). Immobilization of Cellulase in Nanofibrous PVA Membranes by Electrospinning. J. Memb. Sci..

[153] Ren G., Xu X., Liu Q., Cheng J., Yuan X., Wu L., Wan Y. (2006). Electrospun Poly(vinyl Alcohol)/Glucose Oxidase Biocomposite Membranes for Biosensor Applications. React. Funct. Polym..

[154] Hilal N., Kochkodan V., Nigmatullin R., Goncharuk V., Al-Khatib L. (2006). Lipase-Immobilized Biocatalytic Membranes for Enzymatic Esterification: Comparison of Various Approaches to Membrane Preparation. J. Memb. Sci..

[155] Sheldon R.A. (2011). Characteristic Features and Biotechnological Applications of Cross-Linked Enzyme Aggregates (CLEAs). Appl. Microbiol. Biotechnol..

[156] Yusdy, Patel S.R., Yap M.G.S., Wang D.I.C. (2009). Immobilization of L-Lactate Dehydrogenase on Magnetic Nanoclusters for Chiral Synthesis of Pharmaceutical Compounds. Biochem. Eng. J..

[157] Wang A., Wang H., Zhu S., Zhou C., Du Z., Shen S. (2008). An Efficient Immobilizing Technique of Penicillin Acylase with Combining Mesocellular Silica Foams Support and P-Benzoquinone Cross Linker. Bioprocess. Biosyst. Eng..

[158] Subramanian A., Kennel S.J., Oden P.I., Jacobson K.B., Woodward J., Doktycz M.J. (1999). Comparison of Techniques for Enzyme Immobilization on Silicon Supports. Enzym. Microb. Technol..

[159] Sheldon R.A. (2007). Cross-Linked Enzyme Aggregates (CLEAs): Stable and Recyclable Biocatalysts. Biochem. Soc. Trans..

[160] Górecka E., Jastrzębska M. (2011). Immobilization Techniques and Biopolymer Carriers. Biotechnol. Food Sci..

[161] Migneault I., Dartiguenave C., Bertrand M.J., Waldron K.C. (2004). Glutaraldehyde: Behavior in Aqueous Solution, Reaction with Proteins, and Application to Enzyme Crosslinking. Biotechniques.

[162] Öztürk B. (2001). Immobilization of Lipase from Candida Rugosa on Hydrophobic and Hydrophilic Supports.

[163] Lee C.K., Au-Duong A.N. (2017). Enzyme Immobilization on Nanoparticles: Recent Applications. Emerg. Areas Bioeng..

[164] Sharifi M., Sohrabi M.J., Hosseinali S.H., Hasan A., Kani P.H., Talaei A.J., Karim A.Y., Nanakali N.M.Q., Salihi A., Aziz F.M. (2020). Enzyme Immobilization onto the Nanomaterials: Application in Enzyme Stability and Prodrug-Activated Cancer Therapy. Int. J. Biol. Macromol..

[165] Park J.M., Kim M., Park H.S., Jang A., Min J., Kim Y.H. (2013). Immobilization of Lysozyme-CLEA onto Electrospun Chitosan Nanofiber for Effective Antibacterial Applications. Int. J. Biol. Macromol..

[166] Sardar M., Roy I., Gupta M.N. (2000). Simultaneous Purification and Immobilization of Aspergillus Niger Xylanase on the Reversibly Soluble Polymer Eudragit(TM) L-100. Enzym. Microb. Technol..

[167] Ho L.F., Li S.Y., Lin S.C., Hsu W.H. (2004). Integrated Enzyme Purification and Immobilization Processes with Immobilized Metal Affinity Adsorbents. Process Biochem..

[168] Shi Q.H., Tian Y., Dong X.Y., Bai S., Sun Y. (2003). Chitosan-Coated Silica Beads as Immobilized Metal Affinity Support for Protein Adsorption. Biochem. Eng. J..

[169] Sardar M., Gupta M.N. (2005). Immobilization of Tomato Pectinase on Con A–Seralose 4B by Bioaffinity Layering. Enzym. Microb. Technol..

[170] Haider T., Husain Q. (2008). Concanavalin A Layered Calcium Alginate-Starch Beads Immobilized Beta Galactosidase as a Therapeutic Agent for Lactose Intolerant Patients. Int. J. Pharm..

[171] Faruque M.A.A., Syduzzaman M., Sarkar J., Bilisik K., Naebe M. (2021). A Review on the Production Methods and Applications of Graphene-Based Materials. Nanomaterials.

[172] Sun L., Yuan G., Gao L., Yang J., Chhowalla M., Gharahcheshmeh M.H., Gleason K.K., Choi Y.S., Hong B.H., Liu Z. (2021). Chemical Vapour Deposition. Nat. Rev. Methods Primers.

[173] Devi M., Rawat S. (2020). A Comprehensive Review of the Pyrolysis Process: From Carbon Nanomaterial Synthesis to Waste Treatment. Oxf. Open Mater. Sci..

[174] Drogowska-Horná K., Frank O., Kalbac M. (2021). Chemical Vapor Deposition (CVD) Growth of Graphene Films. Graphene: Properties, Preparation, Characterization and Applications.

[175] Chen X., Qu Z., Liu Z., Ren G. (2022). Mechanism of Oxidization of Graphite to Graphene Oxide by the Hummers Method. ACS Omega.

[176] Chen C.H., Hu S., Shih J.F., Yang C.Y., Luo Y.W., Jhang R.H., Chiang C.M., Hung Y.J. (2017). Effective Synthesis of Highly Oxidized Graphene Oxide That Enables Wafer-Scale Nanopatterning: Preformed Acidic Oxidizing Medium Approach. Sci. Rep..

[177] Bakhshandeh R., Shafiekhani A. (2018). Ultrasonic Waves and Temperature Effects on Graphene Structure Fabricated by Electrochemical Exfoliation Method. Mater. Chem. Phys..

[178] Hong G., Han Y., Schutzius T.M., Wang Y., Pan Y., Hu M., Jie J., Sharma C.S., Müller U., Poulikakos D. (2016). On the Mechanism of Hydrophilicity of Graphene. Nano Lett..

[179] Oliveira A.E.F., Braga G.B., Tarley C.R.T., Pereira A.C. (2018). Thermally Reduced Graphene Oxide: Synthesis, Studies and Characterization. J. Mater. Sci..

[180] Some S., Kim Y., Yoon Y., Yoo H., Lee S., Park Y., Lee H. (2013). High-Quality Reduced Graphene Oxide by a Dual-Function Chemical Reduction and Healing Process. Sci. Rep..

[181] Hidayat R., Wahyuningsih S., Ramelan A.H. (2020). Simple Synthesis of RGO (Reduced Graphene Oxide) by Thermal Reduction of GO (Graphene Oxide). IOP Conf. Ser. Mater. Sci. Eng..

[182] Guex L.G., Sacchi B., Peuvot K.F., Andersson R.L., Pourrahimi A.M., Ström V., Farris S., Olsson R.T. (2017). Experimental Review: Chemical Reduction of Graphene Oxide (GO) to Reduced Graphene Oxide (RGO) by Aqueous Chemistry. Nanoscale.

[183] Lesiak B., Trykowski G., Tóth J., Biniak S., Kövér L., Rangam N., Stobinski L., Malolepszy A. (2021). Chemical and Structural Properties of Reduced Graphene Oxide—Dependence on the Reducing Agent. J. Mater. Sci..

[184] Du W., Geng H., Yang Y., Zhang Y., Rui X., Li C.C. (2019). Pristine Graphene for Advanced Electrochemical Energy Applications. J. Power Sources.

[185] Balci E., Yavas B., Goller G. (2021). Investigation of the Effects of Varying Amount of Graphene Nanoplatelets’ (GNPs) Addition on Carbon Nanotubes (CNTs) Reinforced Boron Carbide Produced by Spark Plasma Sintering. J. Aust. Ceram. Soc..

[186] Zhang Y., Wu C., Zhang J. (2013). Interactions of Graphene and Graphene Oxide with Proteins and Peptides. Nanotechnol. Rev..

[187] Al-Qadri A.A.Q., Drmosh Q.A., Onaizi S.A. (2022). Enhancement of Bisphenol a Removal from Wastewater via the Covalent Functionalization of Graphene Oxide with Short Amine Molecules. Case Stud. Chem. Environ. Eng..

[188] Zhang C., Chen S., Alvarez P.J.J., Chen W. (2015). Reduced Graphene Oxide Enhances Horseradish Peroxidase Stability by Serving as Radical Scavenger and Redox Mediator. Carbon.

[189] Pavlidis I.V., Vorhaben T., Tsoufis T., Rudolf P., Bornscheuer U.T., Gournis D., Stamatis H. (2012). Development of Effective Nanobiocatalytic Systems through the Immobilization of Hydrolases on Functionalized Carbon-Based Nanomaterials. Bioresour. Technol..

[190] Tseng C.W., Liao C.Y., Sun Y., Peng C.C., Tzen J.T.C., Guo R.T., Liu J.R. (2014). Immobilization of Clostridium Cellulolyticum D-Psicose 3-Epimerase on Artificial Oil Bodies. J. Agric. Food Chem..

[191] Lee K.H., Lee B., Hwang S.J., Lee J.U., Cheong H., Kwon O.S., Shin K., Hur N.H. (2014). Large Scale Production of Highly Conductive Reduced Graphene Oxide Sheets by a Solvent-Free Low Temperature Reduction. Carbon.

[192] Li W., Wen H., Shi Q., Zheng G. (2016). Study on Immobilization of (+) γ-Lactamase Using a New Type of Epoxy Graphene Oxide Carrier. Process Biochem..

[193] Hernández-Cancel G., Suazo-Dávila D., Ojeda-Cruzado A.J., García-Torres D., Cabrera C.R., Griebenow K. (2015). Graphene Oxide as a Protein Matrix: Influence on Protein Biophysical Properties. J. Nanobiotechnol..

[194] Besharati Vineh M., Saboury A.A., Poostchi A.A., Rashidi A.M., Parivar K. (2018). Stability and Activity Improvement of Horseradish Peroxidase by Covalent Immobilization on Functionalized Reduced Graphene Oxide and Biodegradation of High Phenol Concentration. Int. J. Biol. Macromol..

[195] Dedania S.R., Patel M.J., Patel D.M., Akhani R.C., Patel D.H. (2017). Immobilization on Graphene Oxide Improves the Thermal Stability and Bioconversion Efficiency of D-Psicose 3-Epimerase for Rare Sugar Production. Enzym. Microb. Technol..

[196] Johnson B.J., Russ Algar W., Malanoski A.P., Ancona M.G., Medintz I.L. (2014). Understanding Enzymatic Acceleration at Nanoparticle Interfaces: Approaches and Challenges. Nano Today.

[197] Ding S., Cargill A.A., Medintz I.L., Claussen J.C. (2015). Increasing the Activity of Immobilized Enzymes with Nanoparticle Conjugation. Curr. Opin. Biotechnol..

[198] Pavlidis I.V., Vorhaben T., Gournis D., Papadopoulos G.K., Bornscheuer U.T., Stamatis H. (2012). Regulation of Catalytic Behaviour of Hydrolases through Interactions with Functionalized Carbon-Based Nanomaterials. J. Nanopart. Res..

[199] Jin L., Yang K., Yao K., Zhang S., Tao H., Lee S.T., Liu Z., Peng R. (2012). Functionalized Graphene Oxide in Enzyme Engineering: A Selective Modulator for Enzyme Activity and Thermostability. ACS Nano.

[200] Wei X.L., Ge Z.Q. (2013). Effect of Graphene Oxide on Conformation and Activity of Catalase. Carbon.

[201] Zhang F., Zheng B., Zhang J., Huang X., Liu H., Guo S., Zhang J. (2010). Horseradish Peroxidase Immobilized on Graphene Oxide: Physical Properties and Applications in Phenolic Compound Removal. J. Phys. Chem. C.

[202] Chang Q., Jiang G., Tang H., Li N., Huang J., Wu L. (2015). Enzymatic Removal of Chlorophenols Using Horseradish Peroxidase Immobilized on Superparamagnetic Fe_3_O_4_/Graphene Oxide Nanocomposite. Chin. J. Catal..

[203] Xu H.M., Sun X.F., Wang S.Y., Song C., Wang S.G. (2018). Development of Laccase/Graphene Oxide Membrane for Enhanced Synthetic Dyes Separation and Degradation. Sep. Purif. Technol..

[204] Wang X., Hou C., Qiu W., Ke Y., Xu Q., Liu X.Y., Lin Y. (2017). Protein-Directed Synthesis of Bifunctional Adsorbent-Catalytic Hemin-Graphene Nanosheets for Highly Efficient Removal of Dye Pollutants via Synergistic Adsorption and Degradation. ACS Appl. Mater. Interfaces.

[205] Begum R., Najeeb J., Sattar A., Naseem K., Irfan A., Al-Sehemi A.G., Farooqi Z.H. (2020). Chemical Reduction of Methylene Blue in the Presence of Nanocatalysts: A Critical Review. Rev. Chem. Eng..

[206] Kumar Sahoo P., Panigrahy B., Thakur D., Bahadur D. (2017). Ice-Templating Synthesis of Macroporous Noble Metal/3D-Graphene Nanocomposites: Their Fluorescence Lifetimes and Catalytic Study. New J. Chem..

[207] Patila M., Kouloumpis A., Gournis D., Rudolf P., Stamatis H. (2016). Laccase-Functionalized Graphene Oxide Assemblies as Efficient Nanobiocatalysts for Oxidation Reactions. Sensors.

[208] Kashefi S., Borghei S.M., Mahmoodi N.M. (2019). Covalently Immobilized Laccase onto Graphene Oxide Nanosheets: Preparation, Characterization, and Biodegradation of Azo Dyes in Colored Wastewater. J. Mol. Liq..

[209] Ariaeenejad S., Motamedi E., Hosseini Salekdeh G. (2021). Application of the Immobilized Enzyme on Magnetic Graphene Oxide Nano-Carrier as a Versatile Bi-Functional Tool for Efficient Removal of Dye from Water. Bioresour. Technol..

[210] Vineh M.B., Saboury A.A., Poostchi A.A., Ghasemi A. (2020). Biodegradation of Phenol and Dyes with Horseradish Peroxidase Covalently Immobilized on Functionalized RGO-SiO_2_ Nanocomposite. Int. J. Biol. Macromol..

[211] Ali M., Husain Q., Sultana S., Ahmad M. (2018). Immobilization of Peroxidase on Polypyrrole-Cellulose-Graphene Oxide Nanocomposite via Non-Covalent Interactions for the Degradation of Reactive Blue 4 Dye. Chemosphere.

[212] Yao L.W., Ahmed Khan F.S., Mubarak N.M., Karri R.R., Khalid M., Walvekar R., Abdullah E.C., Mazari S.A., Ahmad A., Dehghani M.H. (2022). Insight into Immobilization Efficiency of Lipase Enzyme as a Biocatalyst on the Graphene Oxide for Adsorption of Azo Dyes from Industrial Wastewater Effluent. J. Mol. Liq..

[213] Yang S., Yang J., Wang T., Li L., Yu S., Jia R., Chen P. (2020). Construction of a Combined Enzyme System of Graphene Oxide and Manganese Peroxidase for Efficient Oxidation of Aromatic Compounds. Nanoscale.

[214] Mahmoodi N.M., Saffar-Dastgerdi M.H. (2020). Clean Laccase Immobilized Nanobiocatalysts (Graphene Oxide—Zeolite Nanocomposites): From Production to Detailed Biocatalytic Degradation of Organic Pollutant. Appl. Catal. B.

[215] Zhu Z., Chen Z., Luo X., Liang W., Li S., He J., Zhang W., Hao T., Yang Z. (2020). Biomimetic Dynamic Membrane (BDM): Fabrication Method and Roles of Carriers and Laccase. Chemosphere.

[216] Lai Y., Wang F., Zhang Y., Ou P., Wu P., Fang Q., Li S., Chen Z. (2019). Effective Removal of Methylene Blue and Orange II by Subsequent Immobilized Laccase Decolorization on Crosslinked Polymethacrylate/Carbon Nanotubes. Mater. Res. Express.

[217] Oliveira S.F., da Luz J.M.R., Kasuya M.C.M., Ladeira L.O., Correa Junior A. (2018). Enzymatic Extract Containing Lignin Peroxidase Immobilized on Carbon Nanotubes: Potential Biocatalyst in Dye Decolourization. Saudi J. Biol. Sci..

[218] Habimana P., Gao J., Mwizerwa J.P., Ndayambaje J.B., Liu H., Luan P., Ma L., Jiang Y. (2021). Improvement of Laccase Activity Via Covalent Immobilization over Mesoporous Silica Coated Magnetic Multiwalled Carbon Nanotubes for the Discoloration of Synthetic Dyes. ACS Omega.

[219] Othman A.M., González-Domínguez E., Sanromán Á., Correa-Duarte M., Moldes D. (2016). Immobilization of Laccase on Functionalized Multiwalled Carbon Nanotube Membranes and Application for Dye Decolorization. RSC Adv..

[220] Jiang S., Ren D., Wang Z., Zhang S., Zhang X., Chen W. (2022). Improved Stability and Promoted Activity of Laccase by One-Pot Encapsulation with Cu (PABA) Nanoarchitectonics and Its Application for Removal of Azo Dyes. Ecotoxicol. Environ. Saf..

[221] Birhanlı E., Noma S.A.A., Boran F., Ulu A., Yeşilada Ö., Ateş B. (2022). Design of Laccase–Metal–Organic Framework Hybrid Constructs for Biocatalytic Removal of Textile Dyes. Chemosphere.

[222] Yang J., Li J., Ng D.H.L., Yang P., Yang W., Liu Y. (2020). Micromotor-Assisted Highly Efficient Fenton Catalysis by a Laccase/Fe-BTC-NiFe_2_O_4_ Nanozyme Hybrid with a 3D Hierarchical Structure. Environ. Sci. Nano.

[223] Ladole M.R., Pokale P.B., Patil S.S., Belokar P.G., Pandit A.B. (2020). Laccase Immobilized Peroxidase Mimicking Magnetic Metal Organic Frameworks for Industrial Dye Degradation. Bioresour. Technol..

[224] Zhang Y., Hu P., Muhammad Y., Tang Y., Shao S., Gao Z., Wang J., Wang R., Hu Y., Kuang L. (2021). High-Density Immobilization of Laccase on Hollow Nano-Sphere NH_2_-MIL88(Fe) Host with Interfacial Defects to Improve Enzyme Activity and Stability for Remazol Brilliant Blue R Decolorization. Chem. Eng. J..

[225] Wang J., Yu S., Feng F., Lu L. (2019). Simultaneous Purification and Immobilization of Laccase on Magnetic Zeolitic Imidazolate Frameworks: Recyclable Biocatalysts with Enhanced Stability for Dye Decolorization. Biochem. Eng. J..

[226] Mahmoodi N.M., Abdi J. (2019). Metal-Organic Framework as a Platform of the Enzyme to Prepare Novel Environmentally Friendly Nanobiocatalyst for Degrading Pollutant in Water. J. Ind. Eng. Chem..

[227] Amari A., Alzahrani F.M., Alsaiari N.S., Katubi K.M., Rebah F.B., Tahoon M.A. (2021). Magnetic Metal Organic Framework Immobilized Laccase for Wastewater Decolorization. Processes.

[228] Chang Q., Huang J., Ding Y., Tang H. (2016). Catalytic Oxidation of Phenol and 2,4-Dichlorophenol by Using Horseradish Peroxidase Immobilized on Graphene Oxide/Fe_3_O_4_. Molecules.

[229] Zhang Q., Xue C., Owens G., Chen Z. (2023). Preparation of Bionanomaterial Based on Green Reduced Graphene Immobilized Ochrobactrum Sp. FJ1: Optimization, Characterization and Its Application. Sep. Purif. Technol..

[230] Costa J.B., Lima M.J., Sampaio M.J., Neves M.C., Faria J.L., Morales-Torres S., Tavares A.P.M., Silva C.G. (2019). Enhanced Biocatalytic Sustainability of Laccase by Immobilization on Functionalized Carbon Nanotubes/Polysulfone Membranes. Chem. Eng. J..

[231] Dai Y., Yao J., Song Y., Wang S., Yuan Y. (2016). Enhanced Adsorption and Degradation of Phenolic Pollutants in Water by Carbon Nanotube Modified Laccase-Carrying Electrospun Fibrous Membranes. Environ. Sci. Nano.

[232] Ren Z., Luo J., Wan Y. (2018). Highly Permeable Biocatalytic Membrane Prepared by 3D Modification: Metal-Organic Frameworks Ameliorate Its Stability for Micropollutants Removal. Chem. Eng. J..

[233] Molina M.A., Díez-Jaén J., Sánchez-Sánchez M., Blanco R.M. (2022). One-Pot Laccase@MOF Biocatalysts Efficiently Remove Bisphenol A from Water. Catal. Today.

[234] Zhang R., Wang L., Han J., Wu J., Li C., Ni L., Wang Y. (2020). Improving Laccase Activity and Stability by HKUST-1 with Cofactor via One-Pot Encapsulation and Its Application for Degradation of Bisphenol A. J. Hazard. Mater..

[235] Rubio-Martinez M., Avci-Camur C., Thornton A.W., Imaz I., Maspoch D., Hill M.R. (2017). New Synthetic Routes towards MOF Production at Scale. Chem. Soc. Rev..

[236] Li G., Pang S., Wu Y., Ouyang J. (2018). Enhanced Removal of Hydroquinone by Graphene Aerogel-Zr-MOF with Immobilized Laccase. Chem. Eng. Commun..

[237] Li D., Cheng Y., Zuo H., Zhang W., Pan G., Fu Y., Wei Q. (2021). Dual-Functional Biocatalytic Membrane Containing Laccase-Embedded Metal-Organic Frameworks for Detection and Degradation of Phenolic Pollutant. J. Colloid Interface Sci..

[238] Wu E., Li Y., Huang Q., Yang Z., Wei A., Hu Q. (2019). Laccase Immobilization on Amino-Functionalized Magnetic Metal Organic Framework for Phenolic Compound Removal. Chemosphere.

[239] Wang D., Lou J., Yuan J., Xu J., Zhu R., Wang Q., Fan X. (2021). Laccase Immobilization on Core-Shell Magnetic Metal-Organic Framework Microspheres for Alkylphenol Ethoxylate Compound Removal. J. Environ. Chem. Eng..

[240] Wu X., Zhang Y., Han T., Wu H., Guo S., Zhang J. (2013). Composite of Graphene Quantum Dots and Fe_3_O_4_ Nanoparticles: Peroxidase Activity and Application in Phenolic Compound Removal. RSC Adv..

[241] Lim X.X., Low S.C., Oh W. (2023). Da A Critical Review of Heterogeneous Catalyst Design for Carbon Nanotubes Synthesis: Functionalities, Performances, and Prospects. Fuel Process. Technol..

[242] Guo T., Nikolaev P., Rinzler A.G., Tomanek D., Colbert D.T., Smalley R.E. (1995). Self-Assembly of Tubular Fullerenes. J. Phys. Chem..

[243] Han F., Qian L., Wu Q., Li D., Hao S., Feng L., Xin L., Yang T., Zhang J., He M. (2022). Narrow-Chirality Distributed Single-Walled Carbon Nanotube Synthesized from Oxide Promoted Fe–SiC Catalyst. Carbon.

[244] Schwandt C., Dimitrov A.T., Fray D.J. (2012). High-Yield Synthesis of Multi-Walled Carbon Nanotubes from Graphite by Molten Salt Electrolysis. Carbon.

[245] Notarianni M., Liu J., Vernon K., Motta N. (2016). Synthesis and Applications of Carbon Nanomaterials for Energy Generation and Storage. Beilstein J. Nanotechnol..

[246] Xu R., Tang R., Zhou Q., Li F., Zhang B. (2015). Enhancement of Catalytic Activity of Immobilized Laccase for Diclofenac Biodegradation by Carbon Nanotubes. Chem. Eng. J..

[247] Chen J., Li Y., Yang Y., Sun H. (2017). How Cushion Communities Are Maintained in Alpine Ecosystems: A Review and Case Study on Alpine Cushion Plant Reproduction. Plant Divers..

[248] Pang R., Li M., Zhang C. (2015). Degradation of Phenolic Compounds by Laccase Immobilized on Carbon Nanomaterials: Diffusional Limitation Investigation. Talanta.

[249] Zhong Z., Pang S., Wu Y., Jiang S., Ouyang J. (2017). Synthesis and Characterization of Mesoporous Cu–MOF for Laccase Immobilization. J. Chem. Technol. Biotechnol..

[250] Ismail U.M., Onaizi S.A., Vohra M.S. (2023). Aqueous Pb(II) Removal Using ZIF-60: Adsorption Studies, Response Surface Methodology and Machine Learning Predictions. Nanomaterials.

[251] Gascón V., Márquez-Álvarez C., Blanco R.M. (2014). Efficient Retention of Laccase by Non-Covalent Immobilization on Amino-Functionalized Ordered Mesoporous Silica. Appl. Catal. A Gen..

[252] Garg A., Jain A. (2014). Hydrogen Storage in Metal-Organic Frameworks: A Review. SGVU Int. J. Environ..

[253] Han Y., Yang H., Guo X., Han Y., Yang H., Guo X. (2020). Synthesis Methods and Crystallization of MOFs.

[254] Han Z., Fan X., Yu S., Li X., Wang S., Lu L. (2022). Metal-Organic Frameworks (MOFs): A Novel Platform for Laccase Immobilization and Application. J. Environ. Chem. Eng..

[255] Silva A.R.M., Alexandre J.Y.N.H., Souza J.E.S., Neto J.G.L., De S., Júnior P.G., Rocha M.V.P., Dos Santos J.C.S., Silva A.R.M., Alexandre J.Y.N.H. (2022). The Chemistry and Applications of Metal–Organic Frameworks (MOFs) as Industrial Enzyme Immobilization Systems. Molecules.

[256] Tao A.R., Habas S., Yang P. (2008). Shape Control of Colloidal Metal Nanocrystals. Small.

[257] Li R., Zhang W., Zhou K., Li R., Zhang W., Zhou K. (2018). Metal–Organic-Framework-Based Catalysts for Photoreduction of CO_2_. Adv. Mater..

[258] Li D.Z., Chen L., Liu G., Yuan Z.Y., Li B.F., Zhang X., Wei J.Q. (2021). Porous Metal–Organic Frameworks for Methane Storage and Capture: Status and Challenges. New Carbon. Mater..

[259] Chuhadiya S., Himanshu, Suthar D., Patel S.L., Dhaka M.S. (2021). Metal Organic Frameworks as Hybrid Porous Materials for Energy Storage and Conversion Devices: A Review. Coord. Chem. Rev..

[260] Chen L., Xu Q. (2019). Metal-Organic Framework Composites for Catalysis. Matter.

[261] Ibrahim A., Vohra M.S., Bahadi S.A., Onaizi S.A., Essa M.H., Mohammed T. (2022). Heavy Metals Adsorption onto Graphene Oxide: Effect of Mixed Systems Anresponse Surface Methodology Modeling. Desalin. Water Treat..

[262] Ganiyu S.A., Awwal Suleiman M., Ahmed Al-Amrani W., Kilaco Usman A., Onaizi S.A. (2023). Adsorptive Removal of Organic Pollutants from Contaminated Waters Using Zeolitic Imidazolate Framework Composites: A Comprehensive and Up-to-Date Review. Sep. Purif. Technol..

[263] Tocco D., Carucci C., Todde D., Shortall K., Otero F., Sanjust E., Magner E., Salis A. (2021). Enzyme Immobilization on Metal Organic Frameworks: Laccase from Aspergillus Sp. Is Better Adapted to ZIF-Zni Rather than Fe-BTC. Colloids Surf. B Biointerfaces.

[264] Pang S., Wu Y., Zhang X., Li B., Ouyang J., Ding M. (2016). Immobilization of Laccase via Adsorption onto Bimodal Mesoporous Zr-MOF. Process Biochem..

[265] Kong X.J., Li J.R. (2021). An Overview of Metal–Organic Frameworks for Green Chemical Engineering. Engineering.

[266] Qin Y., Wan Y., Guo J., Zhao M. (2022). Two-Dimensional Metal-Organic Framework Nanosheet Composites: Preparations and Applications. Chin. Chem. Lett..

[267] Qi L., Luo Z., Lu X. (2019). Biomimetic Mineralization Inducing Lipase-Metal-Organic Framework Nanocomposite for Pickering Interfacial Biocatalytic System. ACS Sustain. Chem. Eng..

[268] Nemiwal M., Gosu V., Zhang T.C., Kumar D. (2021). Metal Organic Frameworks as Electrocatalysts: Hydrogen Evolution Reactions and Overall Water Splitting. Int. J. Hydrogen Energy.

[269] Konnerth H., Matsagar B.M., Chen S.S., Prechtl M.H.G., Shieh F.K., Wu K.C.W. (2020). Metal-Organic Framework (MOF)-Derived Catalysts for Fine Chemical Production. Coord. Chem. Rev..

[270] Lin C., Xu K., Zheng R., Zheng Y. (2019). Immobilization of Amidase into a Magnetic Hierarchically Porous Metal–Organic Framework for Efficient Biocatalysis. Chem. Commun..

[271] He J., Sun S., Zhou Z., Yuan Q., Liu Y., Liang H. (2019). Thermostable Enzyme-Immobilized Magnetic Responsive Ni-Based Metal–Organic Framework Nanorods as Recyclable Biocatalysts for Efficient Biosynthesis of S-Adenosylmethionine. Dalton Trans..

[272] Feng Y., Hu H., Wang Z., Du Y., Zhong L., Zhang C., Jiang Y., Jia S., Cui J. (2021). Three-Dimensional Ordered Magnetic Macroporous Metal-Organic Frameworks for Enzyme Immobilization. J. Colloid Interface Sci..

[273] Tranchemontagne D.J., Tranchemontagne J.L., O’keeffe M., Yaghi O.M. (2009). Secondary Building Units, Nets and Bonding in the Chemistry of Metal-Organic Frameworks. Chem. Soc. Rev..

[274] Chen K., Wu C. (2019). De Designed Fabrication of Biomimetic Metal–Organic Frameworks for Catalytic Applications. Coord. Chem. Rev..

[275] Farmakes J., Schuster I., Overby A., Alhalhooly L., Lenertz M., Li Q., Ugrinov A., Choi Y., Pan Y., Yang Z. (2020). Enzyme Immobilization on Graphite Oxide (GO) Surface via One-Pot Synthesis of GO/Metal-Organic Framework Composites for Large-Substrate Biocatalysis. ACS Appl. Mater. Interfaces.

[276] Gkaniatsou E., Sicard C., Ricoux R., Benahmed L., Bourdreux F., Zhang Q., Serre C., ean-Pierre Mahy J., Steunou N., Gkaniatsou E. (2018). Enzyme Encapsulation in Mesoporous Metal–Organic Frameworks for Selective Biodegradation of Harmful Dye Molecules. Angew. Chem. Int. Ed..

[277] Cui J., Feng Y., Jia S. (2018). Silica Encapsulated Catalase@metal-Organic Framework Composite: A Highly Stable and Recyclable Biocatalyst. Chem. Eng. J..

[278] Uddin M.J., Ampiaw R.E., Lee W. (2021). Adsorptive Removal of Dyes from Wastewater Using a Metal-Organic Framework: A Review. Chemosphere.

[279] Peng J., Wu E., Lou X., Deng Q., Hou X., Lv C., Hu Q. (2021). Anthraquinone Removal by a Metal-Organic Framework/Polyvinyl Alcohol Cryogel-Immobilized Laccase: Effect and Mechanism Exploration. Chem. Eng. J..

[280] Unuofin J.O. (2020). Sustainability Potentials of Novel Laccase Tinctures from Stenotrophomonas Maltophilia BIJ16 and Bordetella Bronchiseptica HSO16: From Dye Decolourization to Denim Bioscouring. Biotechnol. Rep..

[281] Singh A., Varghese L.M., Battan B., Patra A.K., Mandhan R.P., Mahajan R. (2021). Environmental Pollution Reducing Strategy for Scouring of Undegummed Sisal Fibers Using Xylanase and Pectinase Enzymes. Bioprocess. Biosyst. Eng..

[282] Mojsov K. (2017). Enzymatic Scouring and Bleaching of Cotton Terry Fabrics—Opportunity of the Improvement on Some Physicochemical and Mechanical Properties of the Fabrics. J. Nat. Fibers.

[283] Tülek A., Yıldırım D., Aydın D., Binay B. (2021). Highly-Stable Madurella Mycetomatis Laccase Immobilized in Silica-Coated ZIF-8 Nanocomposites for Environmentally Friendly Cotton Bleaching Process. Colloids Surf. B Biointerfaces.

[284] Greca S.C.d.A., Kyrou I., Pink R., Randeva H., Grammatopoulos D., Silva E., Karteris E. (2020). Involvement of the Endocrine-Disrupting Chemical Bisphenol A (BPA) in Human Placentation. J. Clin. Med..

[285] Ohore O.E., Songhe Z. (2019). Endocrine Disrupting Effects of Bisphenol A Exposure and Recent Advances on Its Removal by Water Treatment Systems. A Review. Sci. Afr..

[286] Bilal M., Iqbal H.M.N., Barceló D. (2019). Mitigation of Bisphenol A Using an Array of Laccase-Based Robust Bio-Catalytic Cues—A Review. Sci. Total Environ..

[287] Cañas A.I., Camarero S. (2010). Laccases and Their Natural Mediators: Biotechnological Tools for Sustainable Eco-Friendly Processes. Biotechnol. Adv..

[288] Wang L., Liu Y., Han J., Liu Y., Lan H., Li C., Wang Y. (2021). Morphology-Dependent Intelligent Biocatalysts with Automatic Functionality Regulation for Activity Enhancement and Controllable Recycling. Chem. Eng. J..

[289] Zdarta J., Meyer A.S., Jesionowski T., Pinelo M. (2019). Multi-Faceted Strategy Based on Enzyme Immobilization with Reactant Adsorption and Membrane Technology for Biocatalytic Removal of Pollutants: A Critical Review. Biotechnol. Adv..

[290] Singh J., Saharan V., Kumar S., Gulati P., Kapoor R.K. (2017). Laccase Grafted Membranes for Advanced Water Filtration Systems: A Green Approach to Water Purification Technology. Crit. Rev. Biotechnol..

[291] Sheldon R.A., Basso A., Brady D. (2021). New Frontiers in Enzyme Immobilisation: Robust Biocatalysts for a Circular Bio-Based Economy. Chem. Soc. Rev..

[292] Sheldon R.A., van Pelt S. (2013). Enzyme Immobilisation in Biocatalysis: Why, What and How. Chem. Soc. Rev..

[293] Di Cosimo R., Mc Auliffe J., Poulose A.J., Bohlmann G. (2013). Industrial Use of Immobilized Enzymes. Chem. Soc. Rev..

[294] Cantone S., Ferrario V., Corici L., Ebert C., Fattor D., Spizzo P., Gardossi L. (2013). Efficient Immobilisation of Industrial Biocatalysts: Criteria and Constraints for the Selection of Organic Polymeric Carriers and Immobilisation Methods. Chem. Soc. Rev..

[295] Zucca P., Fernandez-Lafuente R., Sanjust E. (2016). Agarose and Its Derivatives as Supports for Enzyme Immobilization. Molecules.

[296] Kulshrestha Y., Husain Q. (2006). Bioaffinity-Based an Inexpensive and High Yield Procedure for the Immobilization of Turnip (*Brassica rapa*) Peroxidase. Biomol. Eng..

[297] Santos J.C.S.D., Barbosa O., Ortiz C., Berenguer-Murcia A., Rodrigues R.C., Fernandez-Lafuente R. (2015). Importance of the Support Properties for Immobilization or Purification of Enzymes. ChemCatChem.

[298] Siar E.H., Zaak H., Kornecki J.F., Zidoune M.N., Barbosa O., Fernandez-Lafuente R. (2017). Stabilization of Ficin Extract by Immobilization on Glyoxyl Agarose. Preliminary Characterization of the Biocatalyst Performance in Hydrolysis of Proteins. Process Biochem..

[299] Siar E.H., Morellon-Sterling R., Zidoune M.N., Fernandez-Lafuente R. (2019). Amination of Ficin Extract to Improve Its Immobilization on Glyoxyl-Agarose: Improved Stability and Activity versus Casein. Int. J. Biol. Macromol..

[300] Marsden S.R., Mestrom L., McMillan D.G.G., Hanefeld U. (2020). Thermodynamically and Kinetically Controlled Reactions in Biocatalysis—From Concepts to Perspectives. ChemCatChem.

[301] Kasche V. (1986). Mechanism and Yields in Enzyme Catalysed Equilibrium and Kinetically Controlled Synthesis of β-Lactam Antibiotics, Peptides and Other Condensation Products. Enzym. Microb. Technol..

[302] Guajardo N., de María P.D. (2021). Production of Bulk Chemicals with Biocatalysis: Drivers and Challenges Reflected in Recent Industrial Granted Patents (2015–2020). Molecules.

[303] Woodley J.M. (2020). Towards the Sustainable Production of Bulk-Chemicals Using Biotechnology. New Biotechnol..

[304] Asmaly H.A., Abussaud B., Ihsanullah, Saleh T.A., Gupta V.K., Atieh M.A. (2015). Ferric Oxide Nanoparticles Decorated Carbon Nanotubes and Carbon Nanofibers: From Synthesis to Enhanced Removal of Phenol. J. Saudi Chem. Soc..

[305] Del Arco J., Alcántara A.R., Fernández-Lafuente R., Fernández-Lucas J. (2021). Magnetic Micro-Macro Biocatalysts Applied to Industrial Bioprocesses. Bioresour. Technol..

[306] Tavano O.L., Berenguer-Murcia A., Secundo F., Fernandez-Lafuente R. (2018). Biotechnological Applications of Proteases in Food Technology. Compr. Rev. Food Sci. Food Saf..

[307] Santi M., Sancineto L., Nascimento V., Azeredo J.B., Orozco E.V.M., Andrade L.H., Gröger H., Santi C. (2021). Flow Biocatalysis: A Challenging Alternative for the Synthesis of APIs and Natural Compounds. Int. J. Mol. Sci..

[308] Benítez-Mateos A.I., Huber C., Nidetzky B., Bolivar J.M., López-Gallego F. (2020). Design of the Enzyme-Carrier Interface to Overcome the O_2_ and NADH Mass Transfer Limitations of an Immobilized Flavin Oxidase. ACS Appl. Mater. Interfaces.

[309] Garcia-Galan C., Berenguer-Murcia Á., Fernandez-Lafuente R., Rodrigues R.C. (2011). Potential of Different Enzyme Immobilization Strategies to Improve Enzyme Performance. Adv. Synth. Catal..

[310] Rocha-Martín J., De Las Rivas B., Muñoz R., Guisán J.M., López-Gallego F. (2012). Rational Co-Immobilization of Bi-Enzyme Cascades on Porous Supports and Their Applications in Bio-Redox Reactions with In Situ Recycling of Soluble Cofactors. ChemCatChem.

[311] López-Gallego F., Acebrón I., Mancheño J.M., Raja S., Lillo M.P., Guisán Seijas J.M. (2012). Directed, Strong, and Reversible Immobilization of Proteins Tagged with a β-Trefoil Lectin Domain: A Simple Method to Immobilize Biomolecules on Plain Agarose Matrixes. Bioconjug. Chem..

[312] Kastantin M., Langdon B.B., Schwartz D.K. (2014). A Bottom-up Approach to Understanding Protein Layer Formation at Solid–Liquid Interfaces. Adv. Colloid Interface Sci..

[313] Souza P.M.P., Carballares D., Lopez-Carrobles N., Gonçalves L.R.B., Lopez-Gallego F., Rodrigues S., Fernandez-Lafuente R. (2021). Enzyme-Support Interactions and Inactivation Conditions Determine Thermomyces Lanuginosus Lipase Inactivation Pathways: Functional and Florescence Studies. Int. J. Biol. Macromol..

[314] Mateo C., Abian O., Fernández-Lorente G., Pedroche J., Fernández-Lafuente R., Guisan J.M., Tam A., Daminati M. (2002). Epoxy Sepabeads: A Novel Epoxy Support for Stabilization of Industrial Enzymes via Very Intense Multipoint Covalent Attachment. Biotechnol. Prog..

[315] Vranish J.N., Ancona M.G., Oh E., Susumu K., Lasarte Aragonés G., Breger J.C., Walper S.A., Medintz I.L. (2018). Enhancing Coupled Enzymatic Activity by Colocalization on Nanoparticle Surfaces: Kinetic Evidence for Directed Channeling of Intermediates. ACS Nano.

[316] Jäger V.D., Lamm R., Kloß R., Kaganovitch E., Grünberger A., Pohl M., Büchs J., Jaeger K.E., Krauss U. (2018). A Synthetic Reaction Cascade Implemented by Colocalization of Two Proteins within Catalytically Active Inclusion Bodies. ACS Synth. Biol..

[317] Velasco-Lozano S., da Silva E.S., Llop J., López-Gallego F. (2018). Sustainable and Continuous Synthesis of Enantiopure L-Amino Acids by Using a Versatile Immobilised Multienzyme System. ChemBioChem.

[318] Dos Santos J.C.S., Rueda N., Barbosa O., Fernández-Sánchez J.F., Medina-Castillo A.L., Ramón-Márquez T., Arias-Martos M.C., Millán-Linares M.C., Pedroche J., Yust M.D.M. (2015). Characterization of Supports Activated with Divinyl Sulfone as a Tool to Immobilize and Stabilize Enzymes via Multipoint Covalent Attachment. Application to Chymotrypsin. RSC Adv..

[319] Andrés-Sanz D., Diamanti E., Di Silvo D., Gurauskis J., López-Gallego F. (2022). Selective Coimmobilization of His-Tagged Enzymes on Yttrium-Stabilized Zirconia-Based Membranes for Continuous Asymmetric Bioreductions. ACS Appl. Mater. Interfaces.

[320] Velasco Abadia A., Herbert K.M., Matavulj V.M., White T.J., Schwartz D.K., Kaar J.L. (2021). Chemically Triggered Changes in Mechanical Properties of Responsive Liquid Crystal Polymer Networks with Immobilized Urease. J. Am. Chem. Soc..

[321] Zeballos N., Diamanti E., Benítez-Mateos A.I., Schmidt-Dannert C., López-Gallego F. (2021). Solid-Phase Assembly of Multienzyme Systems into Artificial Cellulosomes. Bioconjug. Chem..

[322] Pedroche J., del Mar Yust M., Mateo C., Fernández-Lafuente R., Girón-Calle J., Alaiz M., Vioque J., Guisán J.M., Millán F. (2007). Effect of the Support and Experimental Conditions in the Intensity of the Multipoint Covalent Attachment of Proteins on Glyoxyl-Agarose Supports: Correlation between Enzyme–Support Linkages and Thermal Stability. Enzym. Microb. Technol..

[323] Bolivar J.M., López-Gallego F. (2020). Characterization and Evaluation of Immobilized Enzymes for Applications in Flow Reactors. Curr. Opin. Green Sustain. Chem..

[324] Bolivar J.M., Eisl I., Nidetzky B. (2016). Advanced Characterization of Immobilized Enzymes as Heterogeneous Biocatalysts. Catal. Today.

[325] Barbosa O., Torres R., Ortiz C., Berenguer-Murcia Á., Rodrigues R.C., Fernandez-Lafuente R. (2013). Heterofunctional Supports in Enzyme Immobilization: From Traditional Immobilization Protocols to Opportunities in Tuning Enzyme Properties. Biomacromolecules.

[326] Rodrigues R.C., Berenguer-Murcia Á., Carballares D., Morellon-Sterling R., Fernandez-Lafuente R. (2021). Stabilization of Enzymes via Immobilization: Multipoint Covalent Attachment and Other Stabilization Strategies. Biotechnol. Adv..

[327] Rocchietti S., Ubiali D., Terreni M., Albertini A.M., Fernández-Lafuente R., Guisán J.M., Pregnolato M. (2004). Immobilization and Stabilization of Recombinant Multimeric Uridine and Purine Nucleoside Phosphorylases from *Bacillus subtilis*. Biomacromolecules.

[328] Ubiali D., Rocchietti S., Scaramozzino F., Terreni M., Albertini A.M., Fernández-Lafuente R., Guisán J.M., Pregnolato M. (2004). Synthesis of 2′-Deoxynucleosides by Transglycosylation with New Immobilized and Stabilized Uridine Phosphorylase and Purine Nucleoside Phosphorylase. Adv. Synth. Catal..

[329] De Santis P., Meyer L.E., Kara S. (2020). The Rise of Continuous Flow Biocatalysis—Fundamentals, Very Recent Developments and Future Perspectives. React. Chem. Eng..

[330] Bolivar J.M., Wiesbauer J., Nidetzky B. (2011). Biotransformations in Microstructured Reactors: More than Flowing with the Stream?. Trends Biotechnol..

[331] Rodrigues R.C., Virgen-Ortíz J.J., dos Santos J.C.S., Berenguer-Murcia Á., Alcantara A.R., Barbosa O., Ortiz C., Fernandez-Lafuente R. (2019). Immobilization of Lipases on Hydrophobic Supports: Immobilization Mechanism, Advantages, Problems, and Solutions. Biotechnol. Adv..

